# Randomised, double-blind, multicentre, mixed-methods, dose-escalation feasibility trial of mirtazapine for better treatment of severe breathlessness in advanced lung disease (BETTER-B feasibility)

**DOI:** 10.1136/thoraxjnl-2019-213879

**Published:** 2020-01-08

**Authors:** Irene J Higginson, Andrew Wilcock, Miriam J Johnson, Sabrina Bajwah, Natasha Lovell, Deokhee Yi, Simon P Hart, Vincent Crosby, Heather Poad, David Currow, Emma Best, Sarah Brown, on behalf of BETTER-B Feasibility Trial Group, Rohit Lal

**Affiliations:** 1 Cicely Saunders Institute of Palliative Care, Policy and Rehabilitation, Kings College London and King's College Hospital NHS Foundation Trust, London, UK; 2 Department of Clinical Oncology, University of Nottingham and Nottingham University Hospitals NHS Trust, Nottingham, UK; 3 Wolfson Palliative Care Research Centre, Hull York Medical School, The University of Hull, Hull, UK; 4 Respiratory Research Group, Hull York Medical School, University of Hull, Castle Hill Hospital, University of Hull, Hull, Kingston upon Hull, UK; 5 Palliative Care, Nottingham University Hospitals NHS Trust, Nottingham, UK; 6 Clinical Trials Research Unit (CTRU), University of Leeds, Leeds, West Yorkshire, UK; 7 Faculty of Heath, University of Technology Sydney, Sydney, New South Wales, Australia

**Keywords:** COPD exacerbations, emphysema, lung cancer, palliative care, psychology, COPD pharmacology, drug reactions, idiopathic pulmonary fibrosis

## Abstract

New treatments are required for severe breathlessness in advanced disease. We conducted a randomised feasibility trial of mirtazapine over 28 days in adults with a modified medical research council breathlessness scale score ≥3. Sixty-four patients were randomised (409 screened), achieving our primary feasibility endpoint of recruitment. Most patients had COPD or interstitial lung disease; 52 (81%) completed the trial. There were no differences between placebo and mirtazapine in tolerability or safety, and blinding was maintained. Worst breathlessness ratings at day 28 (primary clinical activity endpoint) were, 7.1 (SD 2.3, placebo) and 6.3 (SD 1.8, mirtazapine). A phase III trial of mirtazapine is indicated. Trial registration: ISRCTN 32236160; European Clinical Trials Database (EudraCT no: 2015-004064-11).

## Introduction

Breathlessness is a prevalent and distressing symptom, associated with considerable disability, social isolation, emergency service use and poor survival.^1 2^ It often persists despite optimum pharmacological treatment of the underlying medical condition and non-drug approaches.^1 3 4^ Drug treatments are limited; opioids have the best evidence,^5 6^ but concerns remain regarding long-term effects. New treatments are required. Antidepressants impact on neurotransmitters involved in various brain circuits potentially affecting breathlessness, and are worthy of consideration.^7^ Data are mixed for selective serotonin reuptake inhibitors, with positive case series but a negative randomised controlled trial.^8 9^ Mirtazapine is an antagonist at α2-adrenergic, H1, 5HT2A/C and 5HT3 receptors, resulting in serotonin, norepinephrine and dopamine release.^7^

Thus, we conducted a multicentre, randomised, placebo-controlled, double-blind, parallel-group, dose-escalating, mixed-methods, feasibility trial of mirtazapine for patients severely affected by breathlessness, to inform a potential phase III trial.

## Methods

For full details, see the Trial Protocol, online supplementary document S1.

10.1136/thoraxjnl-2019-213879.supp1 Supplementary data

### Participants

Patients were recruited from three centres. Inclusion criteria were: consenting adults with a confirmed diagnosis (by hospitals/clinicians) of COPD, interstitial lung disease (ILD), cancer or chronic heart failure, plus clinician assessed modified medical research council (mMRC) breathlessness score of 3 or 4 despite optimal treatment of underlying disease(s) and prognosis of ≥2 months. Main exclusion criteria were: existing antidepressant use and contraindications to mirtazapine.

### Trial design and procedures

Participants were randomised (1:1) to receive oral mirtazapine (15 mg/day (evening)) or placebo (capsules identical in appearance, smell and taste) for 28 days. Randomisation was stratified by disease (cancer vs non-cancer), Hospital Anxiety and Depression Scale (HADS) score (≥15 vs <15), and taking opioids (yes vs no).

The primary endpoint was number of patients recruited across three hospitals over 12 months, with a target of 60. Secondary endpoints, including proposed primary and secondary clinical activity outcomes for a main trial, are in online supplementary box S1. Assessments were at baseline and weekly thereafter, and included evaluation of breathlessness and related activity scales, toxicity, treatment adherence and quality of life. At 14 days, if the rating of worst breathlessness during the previous 24 hours had not improved ≥1 point on the 0–10 numerical rating scale (NRS) over baseline, the daily dose was increased to two capsules (placebo or 30 mg mirtazapine).

Semi-structured qualitative interviews with a purposive sample of participants, aiming to include a mix of diseases, experiences and backgrounds (subject to data saturation), explored motivations for trial participation and experiences of the intervention, procedures and study measures (see Trial Protocol, online supplementary document S1 page 77).

The trial received appropriate approvals from the Medicines and Healthcare products Regulatory Agency, London Central Research Ethics Committee (16/LO/0091), local research governance and registrations; International Standard Randomised Controlled Trial (32236160); EU Clinical Trials Register (2015-004064-11); adopted onto the National Institute for Health Research portfolio (30471).

### Analysis

For the statistical analysis of the primary endpoint (feasibility), we predetermined 60 patients had to be recruited over 12 months across the centres. This sample size took into account the likely number required for a fully powered phase III trial, guidance on feasibility designs and number needed to estimate the overall SD for the phase III primary outcome of worst breathlessness.^10^ As a feasibility trial, all quantitative endpoints were summarised descriptively, with no formal statistical comparisons between groups.

Qualitative data were audio recorded, transcribed verbatim and analysed following the framework method (see online supplementary document S1).

## Results

### Recruitment and progress through trial

**Figure 1:**
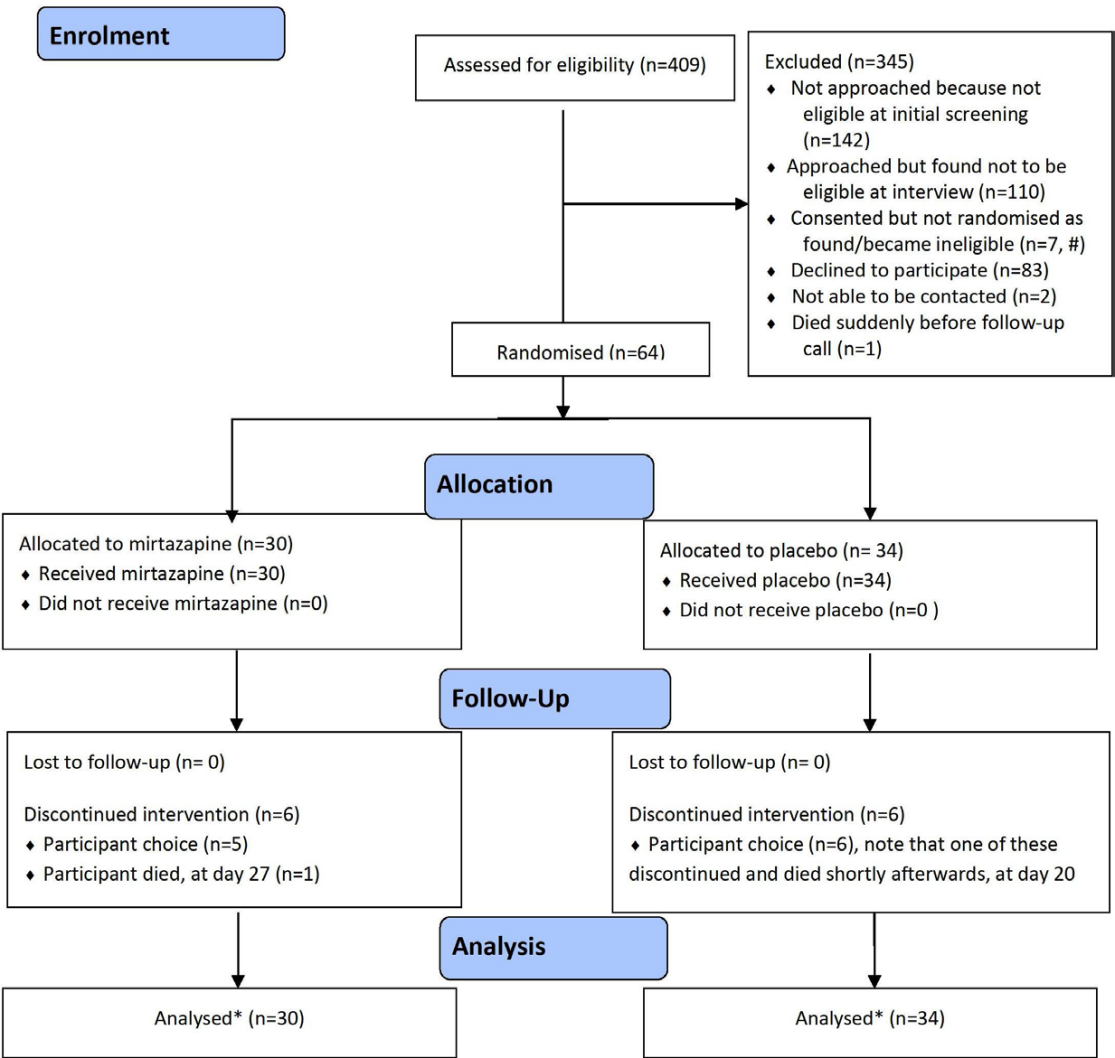
Consolidated Standards of Reporting Trials flow diagram of patients included in the trial, follow-up and analysis. #=reasons why seven patients were consented but not randomised were because they were found or became ineligible: started pulmonary rehabilitation (1); uncontrolled diabetes mellitus (2), started taking antidepressants (1), hepatic impairment (1), decided not on optimal treatment for underlying condition (1), and one missing. * Of those who discontinued intervention, patients were willing to continue data collection in all but one in the mirtazapine group and all but four in the placebo group, all available data were analysed.

Each centre opened for 12 months; 409 patients were screened for eligibility and 64 randomised (16% of those screened; mean 5.3 per month) achieving the primary outcome of feasibility (figure 1). Most participants had COPD (64%) or ILD (31%), and mMRC grade 4 (58%); 33% were taking opioids and HADS score was ≥15 in 24 (38%). Demographics and clinical characteristics were balanced between randomised groups (online supplementary tables S1, S2).

Main reasons for ineligibility were existing antidepressant use (38%), mMRC score <3 (27%). Eighty-three (20% of 409 screened) patients declined participation. Reasons were mainly not liking the idea of a clinical (18%) or a blinded (7%) trial, not wanting to take additional medicine (18%), already having too much to think about (17%) and not liking the thought of antidepressants (13%).

All randomised participants received at least one dose of mirtazapine or placebo, none were lost to follow-up (figure 1). Fifty-two participants (81%) remained on treatment all 28 days. Twenty-nine participants (45%) dose escalated at day 14; based on the same criteria at day 28, 21 participants (33%) would have been eligible for further dose escalation.

Twelve patients (six per arm) discontinued treatment prematurely. The Bang Blinding Index (BBI), which calculates differences between correct and incorrect guesses for unblinding (see online supplementary box S1), was 0.31 (95% CI 0.03 to 0.59) and 0.21 (95% CI −0.07 to 0.50) in the placebo and mirtazapine arms, respectively.

### Toxicity and safety

There were few adverse events, with only one grade 3 reported (insomnia, day 28, placebo arm). There were 12 serious adverse events (SAEs) in nine participants (mean 1.3 per person, SD 0.71; mirtazapine: four people (seven events), placebo: five people (five events)). Only one SAE (a fall, with grade 2 dizziness and confusion) was assessed as being related to trial medication (placebo arm). Two patients died during the study, after 27 (mirtazapine) and 20 (placebo) days from baseline, both due to their underlying illness.

### Other outcome data

**Figure 2:**
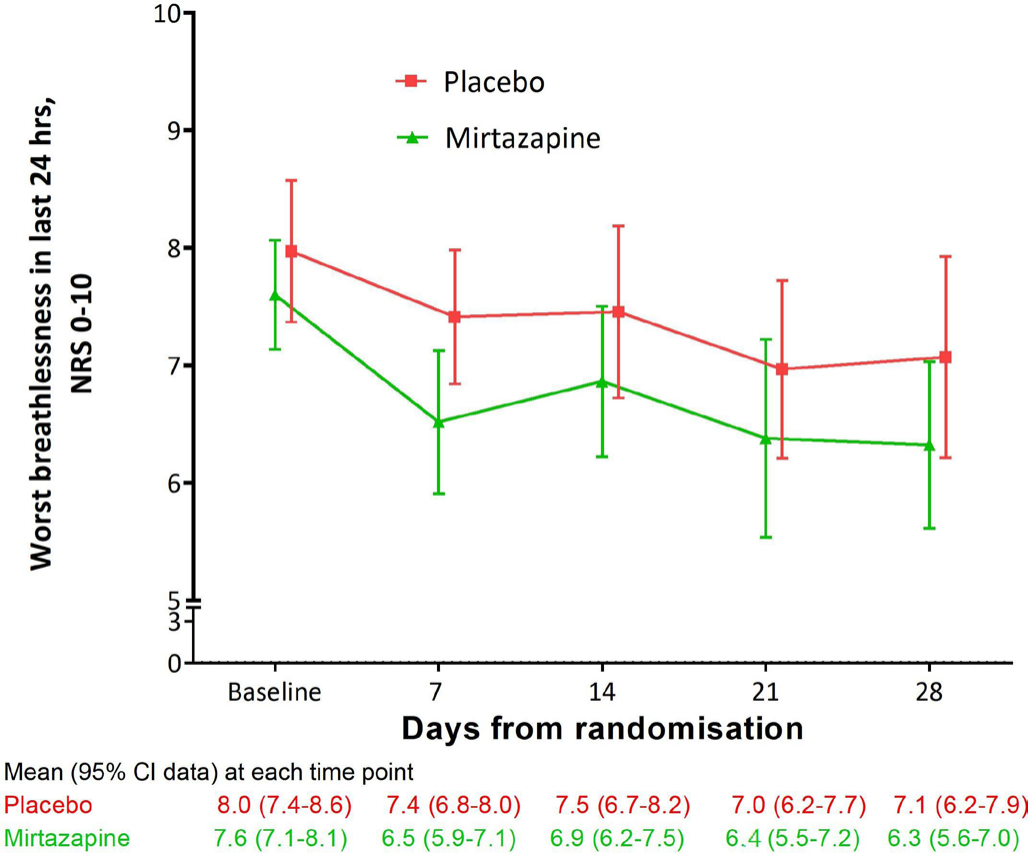
Mean (95% CI) breathlessness at worst and average over 24 hours during the 28 days of the study, by study arm.

There were little missing data (online supplementary table S3). At baseline, mean scores for *worst breathlessness* NRS were similar across treatment arms, with SD of 1.52 informing the phase III sample size calculation. Worst breathlessness improved in both groups at day 7, staying similar subsequently (figure 2). Other outcomes also showed small changes over time (table 1, online supplementary figure S1).

**Table 1:** Clinical activity outcomes and costs at day 28 of study by trial arm

	Mirtazapine n=30	Placebo n=34
**Clinical activity outcomes—mean (SD**)		
NRS worst (last 24 hours)* day 28 change from baseline to day 28 mean (95% CI)	6.3 (1.8) –1.3 (–2.1 to −0.5)	7.1 (2.3) –0.8 (–1.6 to 0.0)
NRS average (last 24 hours)* day 28 change from baseline to day 28 mean (95% CI)	4.7 (2.0) –0.7 (–1.2 to −0.2)	4.9 (1.8) 0.0 (–0.7 to 0.7)
IPOS total* day 28	17.2 (8.0)	17.8 (7.6)
HADS anxiety* day 28 [HADS depression* day 28]	4.3 (2.8) [6.1 (3.3)]	5.3 (3.5) [6.5 (3.7)]
SPPB†‡ day 28	7.6 (2.1)	7.4 (2.9)
CRQ dyspnoea†§¶ day 28 [CRQ emotion†§ day 28]	3.1 (1.1) [5.0 (1.2)]	2.8 (1.0) [4.9 (1.3)]
CRQ fatigue†§ day 28 [CRQ mastery†§ day 28]	3.8 (1.3) [4.9 (1.2)]	4.0 (1.2) [4.9 (1.3)]
GSES total score† day 28	31.4 (5.1)	30.7 (4.8)
**Economic measures—mean (SD**)		
EQ-5D† day 28 change from baseline to day 28 mean (95% CI)	0.61 (0.26) 0.07 (0.00 to 0.15)	0.63 (0.15) 0.03 (–0.04 to 0.09)
EQ-5D VAS† day 28	63.4 (21.2)	60.8 (19.0)
Health and social care costs (£) in the previous 1 month*	522 (773)	412 (529)

*Scale interpretation: high score worse.

†Scale interpretation: high score better.

‡Missing data were higher for SPPB than other measures (see online supplementary table S3)

§CRQ subdomains averaged on the 1–7 scale to give comparability across subscales.

¶Not all patients completed all five activity subscales. However, scores were similar, the data for those completing all five activity subscales are provided here.

CRQ, Chronic Respiratory Disease Questionnaire; EQ-5D, quality of life; GSES, General Self-Efficacy Scale; HADS, Hospital Anxiety and Depression Scale; HRQL, health-related quality of life; IPOS, Integrated Palliative care Outcome Scale; NRS, numerical rating scale/10; SPPB, Short Physical Performance Battery.

### Qualitative data

Interviews were conducted with 22 participants (11 COPD, 8 ILD, 3 other) of whom two withdrew early because of adverse effects, see online supplementary box S2 for summary findings.

## Discussion

This feasibility trial successfully achieved its primary endpoint based on numbers recruited from three sites over 1 year, a pragmatic outcome based on completing a phase III study in a reasonable time. Uptake and data collection were high and attrition low for a population with advanced disease.^3 5 9^ Qualitative data suggest this is partly due to having dedicated research staff. The tolerability and safety of mirtazapine were good with little apparent loss of participant blinding (BBI close to zero in both arms). Our data have been used to inform development of an international multicentre phase III trial; this has secured funding.

## References

[1] Maddocks M, Lovell N, Booth S, et al Palliative care and management of troublesome symptoms for people with chronic obstructive pulmonary disease. The Lancet 2017;390:988–1002. 10.1016/S0140-6736(17)32127-X 28872031

[2] Gysels MH, Higginson IJ The lived experience of breathlessness and its implications for care: a qualitative comparison in cancer, COPD, heart failure and MND. BMC Palliat Care 2011;10:15 10.1186/1472-684X-10-15 22004467 PMC3206451

[3] Higginson IJ, Bausewein C, Reilly CC, et al An integrated palliative and respiratory care service for patients with advanced disease and refractory breathlessness: a randomised controlled trial. The Lancet Respiratory Medicine 2014;2:979–87. 10.1016/S2213-2600(14)70226-7 25465642

[4] Brighton LJ, Miller S, Farquhar M, et al Holistic services for people with advanced disease and chronic breathlessness: a systematic review and meta-analysis. Thorax 2019;74:270–81. 10.1136/thoraxjnl-2018-211589 30498004 PMC6467249

[5] Ekström M, Bajwah S, Bland JM, et al One evidence base; three stories: do opioids relieve chronic breathlessness? Thorax 2018;73:88–90. 10.1136/thoraxjnl-2016-209868 28377491

[6] Simon ST, Higginson IJ, Booth S, et al Benzodiazepines for the relief of breathlessness in advanced malignant and non-malignant diseases in adults. Cochrane Database Syst Rev 2016;10. 10.1002/14651858.CD007354.pub3 PMC6464146 27764523

[7] Lovell N, Wilcock A, Bajwah S, et al Mirtazapine for chronic breathlessness? A review of mechanistic insights and therapeutic potential. Expert Rev Respir Med 2019;13:173–80. 10.1080/17476348.2019.1563486 30596298

[8] Lovell N, Bajwah S, Maddocks M, et al Use of mirtazapine in patients with chronic breathlessness: a case series. Palliat Med 2018;32:1518–21. 10.1177/0269216318787450 30028237

[9] Currow DC, Ekström M, Louw S, et al Sertraline in symptomatic chronic breathlessness: a double blind, randomised trial. Eur Respir J 2019;53 10.1183/13993003.01270-2018 30361250

[10] Browne RH On the use of a pilot sample for sample size determination. Stat Med 1995;14:1933–40. 10.1002/sim.4780141709 8532986

