# Supplementary material for: Randomised, double-blind, multicentre, mixed-methods, dose-escalation feasibility trial of mirtazapine for better treatment of severe breathlessness in advanced lung disease (BETTER-B feasibility)
Source: Thorax. 2020 Jan 8;75(2):176–9. doi: 10.1136/thoraxjnl-2019-213879 (PMC7029228; doi:10.1136/thoraxjnl-2019-213879)
Supplement: Supplementary data [file thoraxjnl-2019-213879supp001.pdf]

Supplementary Material

Randomised, double-blind, multi-centre, mixed-methods, dose-escalation feasibility trial of mirtazapine for better treatment of severe breathlessness in advanced lung disease (BETTER-B Feasibility)

Higginson IJ[1], Wilcock A[2], Johnson M[3], Bajwah S[1], Lovell N[1], Yi D[1], Hart SP[3], Crosby V[2], Poad H[4], Currow DC[3,5], Best E[4], Brown, SR[4] on behalf of BETTER-B Feasibility Trial Group

Table of Contents

Online Document S1- BETTER-B Feasibility Protocol……………………………………………………………………………….2

Online Box S1- Primary, secondary, clinical activity and other endpoints assessed in the BETTER-B Trial…..81

Online Table S1- Baseline demographic, clinical and minimisation characteristics…………………………………….82

Online Table S2- Screening outcomes by method and diagnosis, and screened to recruitment ratio………..83

Online Figure S1- Mean (95% confidence interval) breathlessness at average over 24 hours during the 28 days of the study, by study arm………84

Online Box S2- Qualitative data relating to analysis…………………………………………………………………………………85

Online Table S3- Missing data in the study, categorised using the MOREcare clasification for missing data in palliative care studies, detailing whether item or missed questionnaire due to death or illness……………….88

Online Document S1– BETTER-B Feasibility Protocol

BETTER-B (Feasibility) Protocol, Version 2.0, 11 February 2016

BETTER-B Feasibility:
BETter TreatmEnts for Refractory Breathlessness

A feasibility study of the use of mirtazapine for refractory breathlessness

EudraCT Number: **2015-004064-11**
ISRCTN Number: **32236160**
REC Reference: **16/LO/0091**

Sponsors: King's College London and King's College Hospital NHS Foundation Trust

Funded by: Marie Curie Cancer Care, Cancer Research UK (Grant Ref: A18859)
Supported by: Yorkshire Cancer Research
National Institute for Health and Care Excellence (NIHR)
Cicely Saunders International

Chief Investigator: Prof Irene J. Higginson, Professor of Palliative Care,
King's College London, Cicely Saunders Institute,
Department of Palliative Care, Policy & Rehabilitation,
Bessemer Road, Denmark Hill, London, SE5 9PJ

The Sponsors and Clinical Trials Research Unit (CTRU) accept no responsibility for the accuracy of content reproduced from this protocol and incorporated into additional documentation developed by collaborating or third party organisations.

BETTER-B (Feasibility) Protocol, Version 2.0, 11 February 2016

Key Contacts

**General Enquiries:**	Email: better-b@leeds.ac.uk
**Randomisation:**	Tel: 0113 343 1486
**Pharmacovigilance:**	Fax: 0113 343 6774

Chief Investigator

Professor Irene Higginson
Professor of Palliative Care
Cicely Saunders Institute
King's College London

Tel: 0207 848 5516
Fax: 0207 848 5517
Email: irene.higginson@kcl.ac.uk

Sponsor Representative

Jackie Pullen
King's Health Partners Clinical Trials Office
Great Maze Pond
London SE1 9RT

Tel: 0207 188 5732
Fax: 0207 188 8330
Email: jackie.pullen@kcl.ac.uk

Trial Management *(for queries regarding site set up and regulatory and governance queries)*

Ms Katie Neville
Senior Trial Coordinator
Clinical Trials Research Unit
University of Leeds

Tel: 0113 343 4108
Fax: 0113 343 0686
Email: k.neville@leeds.ac.uk

Mrs Victoria Hiley
Head of Trial Management - Solid Tumours
Clinical Trials Research Unit
University of Leeds

Tel: 0113 343 2657
Fax: 0113 343 0686
Email: v.hiley@leeds.ac.uk

Data Management *(for queries regarding eligibility, treatment, CRF and data query completion, SAE and SUSAR reporting and randomisation process)*

Ms Vicky Liversedge
Trial Coordinator
Clinical Trials Research Unit
University of Leeds

Tel: 0113 343 1486
Fax: 0113 343 6774
Email: v.liversedge@leeds.ac.uk

Statistics

Dr Sarah Brown

Tel: 0113 343 1472

Principal Statistician
Clinical Trials Research Unit
University of Leeds

Fax: 0113 343 1471
Email: s,brown@leeds.ac.uk

Mrs Avie-Lee Tillotson
Medical Statistician
Clinical Trials Research Unit
University of Leeds

Tel: 0113 343 0217
Fax: 0113 343 1471
Email: a.coney@leeds.ac.uk

Trial Physician and Qualitative Interview Sub-study Researcher

Dr Natasha Lovell
Academic Clinical Fellow
Cicely Saunders Institute
King's College London

Tel: 0207 848 5825
Email: natasha.lovell@kcl.ac.uk

Clinical Advisors

Prof Miriam Johnson
Professor of Palliative Medicine, York Medical School, University of Hull and Honorary Consultant to St Catherine's Hospice, Scarborough

Dr Andrew Wilcock
Macmillan Clinical Reader in Palliative Medicine and Medical Oncology, Hayward House Macmillan Specialist Care Cancer Unit, Nottingham City Hospital

Prof David Currow
Professor of Palliative and Supportive Services, Flinders University, Adelaide, Australia

Health Economics Advisor

Dr DeokHee Yi
Health Economist, Cicely Saunders Institute, King's College London

Patient Advisor

Mandy Paine
National Council for Palliative Care

Contents

Trial Summary ... 9
Trial Schema ... 11
Abbreviations ... 12
Background ... 14
1.1 Breathlessness ... 14
1.2 Treatments ... 14
Opioids, oxygen and benzodiazepines ... 14
Antidepressants ... 15
Mirtazapine ... 15
1.3 Current Research and Rationale for BETTER-B Feasibility Trial ... 16
1.4 Justification for Double-Blind Feasibility Trial ... 17
Aims and Objectives ... 17
1.5 Aims ... 17
1.6 Primary Objective ... 17
1.7 Secondary Objectives ... 18
1.7.1 Feasibility ... 18
1.7.2 Activity, Quality of Life and Toxicity ... 18
1.7.3 Qualitative Interview Sub-study ... 19
Design ... 19
Eligibility ... 19
1.8 Inclusion Criteria ... 20
1.9 Exclusion Criteria ... 20
Recruitment Process ... 21
1.10 Recruitment Setting ... 21
1.11 Screening ... 22
1.12 Informed Consent and Eligibility ... 22
1.12.1 Initial Information and Initial Approach ... 23
1.12.2 Consent Process ... 24
1.12.3 Eligibility Process ... 24
1.13 Randomisation ... 26
1.13.1 Timing of Randomisation ... 26
1.13.2 Treatment Allocation ... 26
1.13.3 Randomisation Process ... 26

Higginson IJ, *et al. Thorax* 2020; 75:176–179. doi: 10.1136/thoraxjnl-2019-213879

1.13.4 Post-Randomisation Actions 27
Trial Medicinal Product Management 28
1.14 GSTFT Manufacture, packaging and Labelling 28
1.15 Supply, Distribution and Storage 29
1.16 Dispensing 29
1.17 Reconciliation 30
BETTER-B Treatment 30
1.18 Treatment Details 30
1.18.1 Treatment Regimen 30
1.18.2 Treatment Compliance 31
1.18.3 Concomitant Medications / Interactions 31
1.18.4 Most frequent anticipated toxicities 32
1.19 Emergency Unblinding 32
1.19.1 Emergency Unblinding during Office Hours 33
1.19.2 Emergency Unblinding outside of Office Hours 33
1.19.3 Treatment of Participants following Emergency Unblinding 34
1.20 Withdrawal of Treatment 34
Assessments and Data Collection 34
1.1 Schedule of Events 34
1.2 Screening Data 36
1.3 Eligibility Assessments 36
1.4 Baseline Assessments and Data Collection 37
1.5 Trial Treatment Assessments and Data Collection 38
1.5.1 Day 7 Assessment Phone Call 38
1.5.2 Day 14 Assessment Visit and potential dose escalation 38
Dose Escalation 39
1.5.3 Day 21 Assessment Phone Call 40
1.5.4 Day 28 Assessment Visit 40
1.6 Follow-up Assessment and Data Collection 41
1.7 End of Trial Treatment 42
1.8 Adverse Events and Serious Adverse Events 42
1.9 Pregnancies 42
1.10 Deaths 42
1.11 Important Medical Events (IMEs) 43

1.12 Protocol Deviations and Violations 43
1.13 End of Trial Definition 43
1.14 Trial Data and Documentation held at sites 43
1.15 Case Report Forms (CRFs) 43
Pharmacovigilance 44
1.16 General Definitions 44
1.17 BETTER-B Operational Definitions 46
1.17.1 Adverse Events (AEs) / Adverse Reactions (ARs) and Serious Adverse Events (SAEs) / Serious Adverse Reactions (SARs) 46
1.17.2 Suspected Unexpected Serious Adverse Reactions (SUSARs) 48
1.18 BETTER-B Reporting Requirements 48
1.18.1 Reporting of Adverse Events (AEs) and Adverse Reactions (ARs) 49
1.18.2 Expedited Reporting of Serious Adverse Events (SAEs), Serious Adverse Reactions (SARs) and Suspected Unexpected Serious Adverse Reactions (SUSARs) 49
1.19 Responsibilities 50
Participant-reported measures 52
Economic Evaluation 53
Endpoints 54
1.20 Primary Endpoint 54
1.21 Secondary Endpoints 54
1.21.1 Feasibility 54
1.21.2 Activity 55
1.21.3 Safety and Toxicity 55
1.21.4 Symptoms and Quality of Life 55
Statistical Considerations 56
Sample size and planned recruitment rates 56
Statistical Analysis 56
1.22 General Considerations 56
1.23 Analysis populations 57
1.24 Frequency of analysis 57
1.25 Primary Endpoint Analysis 57
1.26 Secondary Endpoint Analysis 58
1.26.1 Feasibility 58
1.26.2 Activity 59
1.26.3 Safety and Toxicity 59

1.26.4 Quality of Life and Patient-reported outcomes 60
Trial Monitoring 60
1.27 Trial Steering Committee 60
1.28 Data Monitoring 61
1.29 Clinical Governance Issues 61
Quality Assurance, Ethical and Regulatory Considerations 61
1.30 Quality Assurance 61
1.31 Serious Breaches 61
1.32 Ethical and Regulatory Considerations 62
Confidentiality 62
Archiving 63
Statement of Indemnity 64
Study Organisational Structure 64
1.33 Individuals and Individual Organisations 64
1.34 Oversight and Trial Monitoring Groups 66
Publication Policy 66
References 68
Appendix A: Modified Medical Research Council (mMRC) Dyspnoea Scale 73
Appendix B: Australia-modified Karnofsky Performance Scale (AKPS) 74
Appendix C: New York Heart Association (NYHA) 75
Appendix D: Qualitative Sub-study 76
1.35 Background 76
1.36 Aim 76
1.37 Patient Interview Eligibility Criteria 77
1.38 Sampling 77
1.39 Consent Process 77
1.40 Interview Procedure 78
1.41 Data analysis 79
1.42 Endpoints 79
1.43 Ethical Considerations 79
1.44 Confidentiality 80

Trial Summary

Trial Title (Full)	BETTER-B (Feasibility): **BET**ter Treatm**E**nts for **R**efractory **B**reathlessness: A feasibility study of the use of mirtazapine for refractory breathlessness
Trial Title (Short)	BETTER-B (Feasibility): **BET**ter Treatm**E**nts for **R**efractory **B**reathlessness
Trial Acronym	BETTER-B (Feasibility)
Trial Background	Breathlessness (also called dyspnea or dyspnoea) is a common, distressing symptom in advanced disease, particularly those affecting the heart and lungs, causing considerable disability for patients, and anxiety and social isolation for them and their family and carers. Breathlessness which continues despite optimal management of the underlying causes and current symptom relief measures, is termed 'intractable' or 'refractory'. It generally worsens as the disease progresses and is one of the most frightening aspects facing a person with advanced disease.<br><br>Over 2 million people experience breathlessness each year in the UK. This includes more than 90% of the over 1 million people in the UK diagnosed with moderate to severe chronic lung disease, over 50% of the 200,000+ with incurable cancer and 50% of the 2 million with chronic heart failure (many of whom will suffer refractory breathlessness). Breathlessness is associated with shortened life expectancy and often results in emergency visits and hospitalisation.<br><br>There are few effective treatments for refractory breathlessness, thus, refractory breathlessness represents a huge unmet need and new approaches are desperately required. Morphine has a role, but there are no other proven pharmacological treatments. Preliminary data suggest that serotonergic modulation is beneficial but rigorous evaluation has not been conducted. There is therefore a need to explore the potential role of antidepressants in this setting.<br>Mirtazapine is a widely used noradrenergic and specific serotonergic antidepressant (NaSSA). There is clinical experience to support its use in anxiety and panic disorder and clinical evidence for its use in major depressive disorders associated with anxiety.<br>BETTER-B (Feasibility) will help address this unmet need by exploring if mirtazapine has a role in the management of refractory breathlessness in patients with cancer, chronic obstructive pulmonary disease, interstitial lung disease, or chronic heart failure. If successful, a larger trial will be conducted.
Trial Design	A randomised (1:1) placebo-controlled, double-blind, mixed-methods, multicentre (3 trial sites) feasibility trial.
Trial Aim	The aim of the study is to determine the feasibility of performing a large-scale double-blind, placebo-controlled randomised trial of mirtazapine for refractory breathlessness.
Trial Endpoints	Primary endpoint:<br>• Number of patients recruited across 3 trial sites over a 12-month period<br>Secondary endpoints:<br>• Feasibility

Higginson IJ, *et al. Thorax* 2020; 75:176–179. doi: 10.1136/thoraxjnl-2019-213879

	• Activity<br>• Safety and toxicity<br>• Symptoms and Quality of Life:<br>*Ancillary study*:<br>• Qualitative Interviews
Trial Population	60 participants suffering from severe refractory breathlessness, diagnosed with cancer, lung disease (COPD/ILD) or chronic heart failure.
Randomisation	Randomisation (1:1) to the placebo arm or the active arm (mirtazapine) will be carried out by the Clinical Trials Research Unit (CTRU), Leeds at baseline prior to starting treatment.
Trial Treatment	All participants are planned to receive 28 days of trial treatment, taking 1 capsule of trial drug (placebo or 15mg mirtazapine) daily.<br>After an initial 14 days of treatment, participants will be assessed for suitability to escalate their dose. If deemed suitable, participants will increase their dose to 2 capsules daily (placebo or 30mg mirtazapine).
Duration	Trial recruitment will continue for 12 months and be followed by a short follow-up period (until the last randomised participant's follow-up assessment).
Evaluation of outcome measures	Participants will be assessed (either by phone or in person) every 7 days during trial treatment (day 7, 21 and 28) and then have a follow-up assessment (by phone) 7 days after their last trial drug dose.

Higginson IJ, *et al. Thorax* 2020; 75:176–179. doi: 10.1136/thoraxjnl-2019-213879

Trial Schema

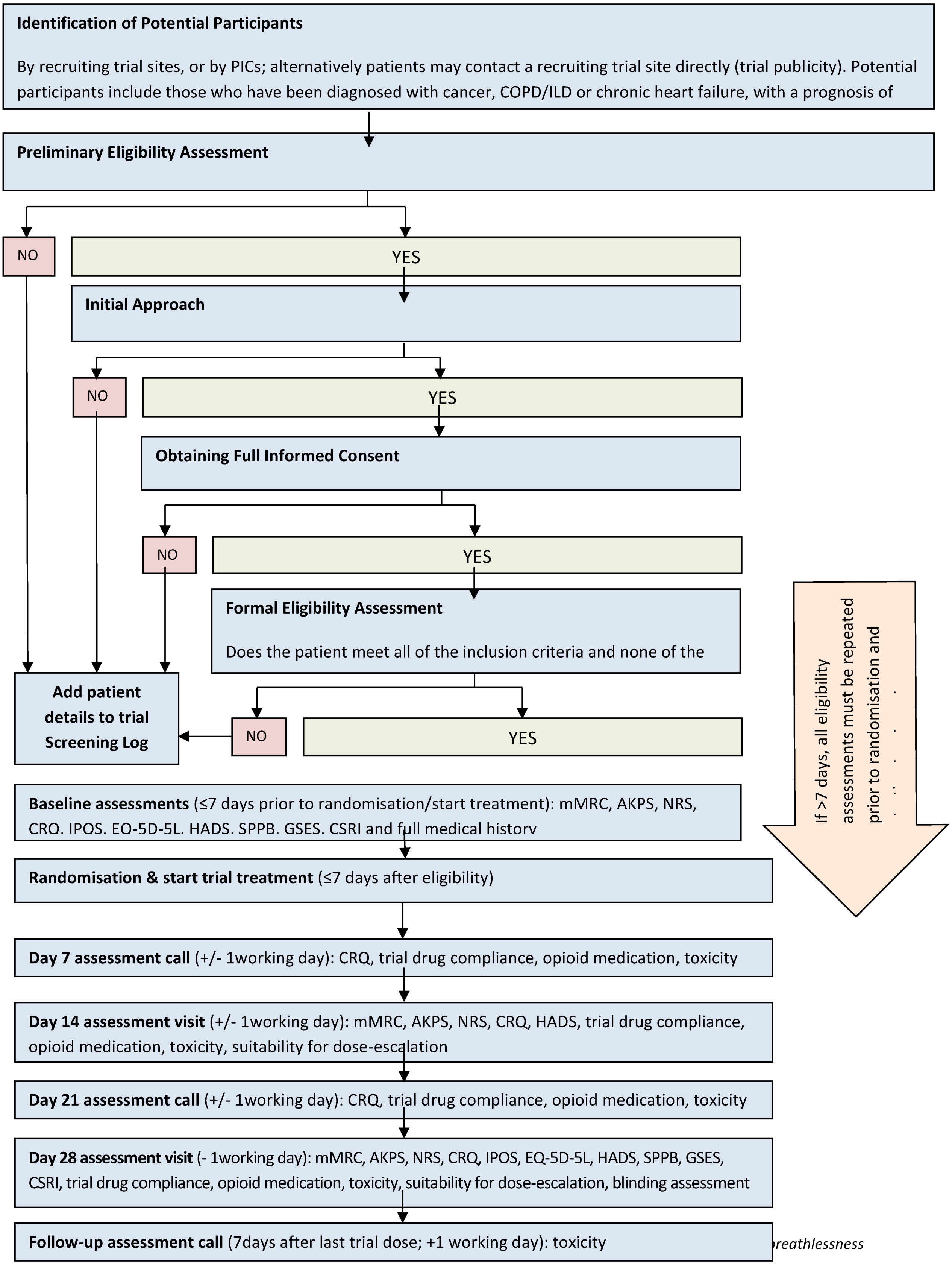

Abbreviations

AE	Adverse Event
AKPS	Australia-modified Karnofsky Performance Scale
AR	Adverse Reaction
ATS	American Thoracic Society
CI	Chief Investigator
COPD	Chronic Obstructive Pulmonary Disease
CRF	Case Report Form
CRQ	Chronic Respiratory Questionnaire
CSRI	Client Services Receipt Inventory
CTA	Clinical Trial Authorisation
CTCAE	Common Terminology Criteria for Adverse Events
CTD	Connective Tissue Disease
CTRU	Clinical Trials Research Unit
DMEC	Data Monitoring & Ethics Committee
DSUR	Development Safety Update Report
ETS	European Respiratory Society
GCP	Good Clinical Practice
GSES	Generalized Self-Efficacy Scale
GSTFT	Guy’s and St Thomas’ NHS Foundation Trust
HADS	Hospital Anxiety and Depression Scale
ICF	Informed Consent Form
ICMJE	International Committee of Medical Journal Editors
ILD	Interstitial Lung Disease
IME	Important Medical Event
IMP	Investigational Medicinal Product
IPF	Idiopathic Pulmonary Fibrosis
IPOS	Integrated Palliative care Outcome Scale
ITT	Intention To Treat
MAO-I	Monoamine Oxidase Inhibitor
MDT	Multi-Disciplinary Teams
MedDRA	Medical Dictionary for Regulatory Activities
MHRA	Medicines and Healthcare products Regulatory Authority
mMRC	modified Medical Research Council
NaSSA	Noradrenergic and Specific Serotonergic Antidepressant
NCI	National Cancer Institute
NCRI	National Cancer Research Institute
NICE	National Institute for Health and Care Excellence
NRS	Numerical Rating Scale
NYHA	New York Heart Association
PI	Principal Investigator
PIC	Participants Identification Centre
PIS	Participant Information Sheet
PSL	Participant Summary Leaflet
QALY	Quality Adjusted Life Year
QoL	Quality of Life
REC	Research Ethics Committee
SAE	Serious Adverse Event
SAR	Serious Adverse Reaction
SOP	Standard Operating Procedure
SPC	Summary of Product Characteristics
SPPB	Short Physical Performance Battery
SSOP	Study Site Operating Procedure
SSRI	Selective Serotonin Re-uptake Inhibitor
SUSAR	Suspected Unexpected Serious Adverse Reaction

Higginson IJ, *et al. Thorax* 2020; 75:176–179. doi: 10.1136/thoraxjnl-2019-213879

TCA	Tricyclic Antidepressant
TMG	Trial Management Group
TSC	Trial Steering Committee

Higginson IJ, *et al*. *Thorax* 2020; 75:176–179. doi: 10.1136/thoraxjnl-2019-213879

Background

1.1 Breathlessness

Breathlessness is a common, distressing symptom in life-limiting conditions, particularly those affecting the heart and lungs, causing considerable disability for patients [1-3], and anxiety and social isolation for them and their family and carers [4-6]. Breathlessness (also called dyspnea or dyspnoea) is usually described as: "a subjective experience of breathing discomfort that consists of qualitatively distinct sensations that vary in intensity. The experience derives from interaction among multiple physiological, psychological, social, and environmental factors and may induce secondary physiological and behavioural responses" [1]. Breathlessness which continues despite optimal management of the underlying causes and current symptom relief measures, is termed 'intractable' or 'refractory'. It generally worsens as the disease progresses and is one of the most frightening aspects facing a person with advanced disease. Caregivers often report a feeling of helplessness while watching their loved ones suffer; clinicians too experience similar feelings due to the lack of effective interventions [4-6].

Over 2 million people experience breathlessness each year in the UK. This includes more than 90% of the over 1 million people in the UK diagnosed with moderate to severe chronic lung disease, over 50% of the 200,000+ with incurable cancer and 50% of the 2 million with chronic heart failure (many of whom will suffer refractory breathlessness) [7-10]. In addition, breathlessness is found in people severely affected by renal and liver failure, neurological conditions, HIV/AIDS and many autoimmune diseases [8, 11-13]. Breathlessness increases as the disease progresses [14, 15], is associated with shortened life expectancy [16-18], is very frightening for patients and families [5, 6, 9, 10, 19] and often results in emergency visits and hospitalisation [3, 20-22].

1.2 Treatments

There are few effective treatments for refractory breathlessness. Morphine has a role [23-29], but there are no other proven pharmacological treatments. Animal studies, case reports, observational series and a phase II trial of 10 patients suggest that serotonergic modulation is beneficial [30-34], but rigorous evaluation has not been conducted.

Opioids, oxygen and benzodiazepines

The most relevant reviews are available for opioids, oxygen and benzodiazepines [29, 35-38]. Although opioids by mouth and injection can reduce breathlessness, optimal dosing, titration and potential issues arising from longer term use (e.g. safety, tolerance, dependence, misuse) remain to be determined. Further, not all patients may be suitable for, or want them [27, 29, 39], especially those with non-malignant disease. In one Dutch study, only 2% of people with advanced Chronic Obstructive Pulmonary Disease (COPD) were prescribed strong opioids [9]. Evidence does not support the use of nebulized opioids or transmucosal fentanyl [29, 38]. Benefit

from oxygen is similar to medical air in mildly or non-hypoxaemic breathless patients with various diseases, and there are limitations to its use (e.g. safety, cost) [40]. The evidence currently does not support a role for benzodiazepines [37]. Thus, the need remains to develop new palliative approaches with fewer limitations.

Antidepressants

Antidepressants are an attractive option to explore, particularly given their low risk of respiratory depression and dependence [30]. There are no systematic reviews relating to such use. Data is limited, but animal work [32] and case series of patients with chronic breathlessness reporting improvement in breathlessness ± exercise tolerance with older tricyclic antidepressants (TCA) or selective serotonin re-uptake inhibitor (SSRI) antidepressants [30, 31, 33], suggest that serotonin plays a role in the control of respiration and generation/perception of breathlessness. The exact mechanism is unclear; a reduction in sensitivity to CO2 has been reported [41]; ultimately it probably involves modulation of brain stem centres responsible for respiratory rhythm and/or of centres involved in the perception of breathlessness [31].

Benefit does not appear to relate to antidepressant or anxiolytic effects *per se*, as improvements in breathlessness are also reported by patients without concurrent anxiety and/or depression [31, 33, 42]. However, manipulation of serotonin in patients with panic disorder reduces experimentally induced panic (using CO2 challenges) and given that 'respiratory anxiety and panic' are common in patients with chronic breathlessness, this could be relevant [41].

Mirtazapine

Mirtazapine is a widely used noradrenergic and specific serotonergic antidepressant (NaSSA). It antagonises receptors (α2*,* 5HT2A and 5HT2C) which inhibit the release of serotonin, noradrenaline and dopamine [43, 44]. In addition, it antagonizes H1- and 5HT3- receptors.

Mirtazapine is a commonly used antidepressant with good data supporting its efficacy, acceptability and safety in the treatment of depressive illness [31, 42, 45]. There is clinical experience to support its use in anxiety and panic disorder and clinical evidence for its use in major depressive disorders associated with anxiety [46].

It is an antidepressant which appears to have a quicker onset of action and fewer drug interactions than other antidepressants, has a good safety record and may be better tolerated than other antidepressants in this population [45-50]. It also has the added advantage of reducing anxiety [47-49, 51], which is a common consequence of severe episodes of breathlessness [52-54].

Mirtazapine is increasingly preferred over SSRIs (and other antidepressants) in advanced disease because it appears to have a quicker onset of action [45] and it has fewer undesirable effects leading to early discontinuation, fewer drug interactions and is not associated with cardiovascular toxicity or sexual dysfunction [55].

Higginson IJ, *et al. Thorax* 2020; 75:176–179. doi: 10.1136/thoraxjnl-2019-213879

Further, it also has appetite stimulation, anti-emetic and analgesic properties, which could be of particular benefit to patients with advanced disease, and the side effect of weight gain may also be an advantage for some patients who have weight loss in advanced disease [50, 51, 56-59]. Thus, mirtazapine is a promising alternative to SSRIs to test in this setting.

1.3 Current Research and Rationale for BETTER-B Feasibility Trial

Despite an increase in the understanding of the mechanisms associated with the generation and perception of breathlessness, this has not yet translated into effective new treatment options [1]. Thus, refractory breathlessness represents a huge unmet need and new approaches are desperately required. Authoritative guidelines have highlighted the need for interdisciplinary research to test new treatments in sufficiently powered clinical trials. They have also stressed the importance of not limiting potentially universally beneficial approaches to one particular patient group [1, 35, 36, 60-62].

There is a need to explore the potential role of antidepressants in this setting. Existing data is limited, but reflects that SSRIs (e.g. sertraline) are now generally preferred over TCAs from a tolerability point of view [50, 55-58]. A search of current trial databases on the management of breathlessness (and dyspnoea), including clinicaltrials.gov and controlled-trials.com, and contact with leaders in the field identified one study of morphine (Johnson), one relevant phase II [34] and one phase III study of sertraline [63] (Currow, personal communication) and no studies of mirtazapine. Currow's randomised trial in Australia is testing sertraline in the management of breathlessness across several conditions following promising phase II data [34, 63]. This trial, recruiting in 12 hospitals, has (as of October 2015) 160 patients randomised (total needed 220), and 107 completed (total needed 150). However, as a partner in this application, the Australian group has raised concerns that because of the many contra-indications for sertraline use, the number of potential drug interactions and undesirable effects, many otherwise eligible patients are excluded from the trial, leading not only to slow recruitment (first patient enrolled January 2011), but more importantly, to concerns that the results may have limited generalisability. Thus, BETTER-B will test a different category of antidepressant in this setting, one which may have advantages over sertraline and other SSRIs.

Preliminary data suggest that serotonergic modulation is beneficial but rigorous evaluation has not been conducted. BETTER-B will help address this need by exploring if mirtazapine has a role in the management of refractory breathlessness in patients with cancer, chronic obstructive pulmonary disease, interstitial lung disease, or chronic heart failure.

Higginson IJ, *et al. Thorax* 2020; 75:176–179. doi: 10.1136/thoraxjnl-2019-213879

1.4 Justification for Double-Blind Feasibility Trial

Breathlessness is a complex, multifactorial experience and is reported as a subjective measure, and refractory breathlessness is a feature of advanced disease where participants may suffer from adverse events due to their underlying condition(s). Therefore, in order to gain a measure of the benefits and harms of an intervention in a trial, a placebo control is needed.

A large-scale trial was considered, however in light of the uncertainty around recruitment, blinding and attrition in this trial, and in order to obtain an estimate of likely activity of this drug to inform the design of the large-scale trial, a feasibility trial was deemed necessary first. This feasibility trial will determine whether a large-scale trial in this advanced illness setting can be performed. The trial is placebo-controlled and double-blind, to reflect exactly the proposed design of a subsequent large-scale trial. A 7-day follow-up period has been chosen in order to provide sufficient data on recruitment and retention for a large-scale trial.

The results of this trial will be used to determine the feasibility of proceeding with a large randomised, placebo-controlled, double-blind trial of approximately 250 patients in this setting and this population and the best methods/design for that study. They will inform future studies in patients with advanced diseases, especially recruitment and trial design, and advance our understanding of breathlessness and ways to research it.

A formal feasibility trial design was not deemed necessary as there is evidence of the activity and safety of other similar drugs in the same class as mirtazapine, providing preliminary evidence that a large-scale trial of mirtazapine is warranted (see section 1.2 and personal confidential communication, Currow et al., Trial Management Group member).

Aims and Objectives

This is a randomised placebo-controlled, double-blind, mixed-methods feasibility trial of mirtazapine for refractory breathlessness in 60 patients with a diagnosis of cancer, chronic obstructive pulmonary disease (COPD), interstitial lung disease (ILD) or chronic heart failure.

1.5 Aims

The aim of the trial is to determine the feasibility of performing a large-scale double-blind, placebo-controlled randomised trial of mirtazapine for refractory breathlessness.

1.6 Primary Objective

The primary objective is to determine whether a randomised, double-blind, placebo-controlled large-scale trial of mirtazapine for refractory breathlessness is feasible in terms of recruitment, as assessed by the number of patients recruited across 3 hospitals over a 12-month period.

1.7 Secondary Objectives

1.7.1 Feasibility

To quantitatively assess a number of other aspects of feasibility, which will be used to determine whether the current design is suitable to be taken forward to a large-scale trial. To be assessed in terms of:

- Recruitment in different settings: outpatient, community services and inpatient settings
- Acceptability of randomisation to patients
- Ability to deliver placebo-control and maintain the double-blind
- Ability to assess outcome measures and minimise missing data for the future large-scale trial
- Compliance with treatment

1.7.2 Activity, Quality of Life and Toxicity

To obtain average and worst breathlessness severity estimates (measured using Numerical Rating Scale (NRS)) on day 28 to inform the sample size calculations for the future large-scale trial.

To determine patient eligibility to increase the dose of mirtazapine further at 28 days.

To assess the potential activity and impact on the activity of mirtazapine and quality of life (QoL) for patients with refractory breathlessness using the following tools:

- Breathlessness mastery: Chronic Respiratory Questionnaire (CRQ) and modified Medical Research Council (mMRC) dyspnoea scale [64] on days 14 and 28;
- Lower extremity functioning: Short Physical Performance Battery (SPPB) [65] on day 28 [66];
- Coping self-belief assessment: Generalized Self-Efficacy Scale (GSES) [67] on day 28;
- Palliative symptoms: Integrated Palliative care Outcome Scale (IPOS) on days 14 and 28;
- Anxiety and depression: Hospital Anxiety and Depression Scale (HADS) on days 14 and 28;
- QoL: EQ-5D-5L on day 28 and Australia-modified Karnofsky Performance Scale (AKPS) [68] on days 14 and 28;
- Health Economics: Client Services Receipt Inventory (CSRI) on day 28;
- Opioid medication: on days 7, 14, 21 and 28;

To monitor adverse reactions, using the Common Terminology Criteria for Adverse Events (CTCAE) categorisation (v4) [69], on days 7, 14, 21 and 28, in order to evaluate the toxicity profile of mirtazapine in patients with refractory breathlessness.

If we are able to demonstrate feasibility within this trial, i.e. the ability to recruit an average of 5 patients per month within a 12-month period (approximately 60 patients) then we plan to seek funding to run a larger double-blind, placebo-controlled randomised trial. Secondary outcome measures of feasibility will be assessed to determine whether the design of the future large-scale trial may need to be adapted to improve recruitment or reduce attrition. Physical activity and toxicity outcomes will be used to inform the design of the future trial however they will not be used to inform the decision as to whether or not to proceed to a larger scale trial. The primary aim of the future trial would be to determine whether mirtazapine improves breathlessness in patients with refractory breathlessness compared to placebo, based on breathlessness severity at day 28 as the primary outcome measure.

1.7.3 Qualitative Interview Sub-study

We will conduct interviews with a sub-set of patients to explore acceptability of trial procedures, materials and intervention for patients and clinicians, and the main impact, if any, of the intervention to enhance recruitment procedures, and ensure that the outcome measures are appropriate for a large-scale trial.

Design

The trial is designed as a multi-centre, randomised, placebo-controlled, double-blind, mixed-methods feasibility trial. It is planned to recruit approximately 60 participants with refractory breathlessness over a 12-month period from approximately three UK trial sites.

Participants will be randomised via minimisation on a 1:1 ratio to receive either oral mirtazapine (15mg/day) or placebo medication for 28 days.

At day 14 of treatment breathlessness intensity ("at worst" over the previous 24 hours) will be assessed using the numerical rating scale (NRS). This will be assessed by a member of the research team. If there is no improvement in NRS (i.e. NRS does not increase by 1 point or more compared to baseline NRS) and the drug has been well tolerated, the daily dose of treatment will be doubled.

Participants will be followed up for 7 days after completing trial treatment to assess safety and toxicity of treatment.

All participants, trial site research teams and pharmacies will be blinded to participants' treatment allocation to minimise possible bias. Further information regarding blinding can be found in section 0.

Eligibility

Eligibility waivers to inclusion and exclusion criteria are not permitted.

Higginson IJ, *et al. Thorax* 2020; 75:176–179. doi: 10.1136/thoraxjnl-2019-213879

1.8 Inclusion Criteria

Male or female aged ≥ 18 years old

Diagnosed with:

- Cancer, or
- Chronic obstructive pulmonary disease (COPD), or
- Interstitial lung disease (ILD), or
- Chronic heart failure (New York Heart Association (NYHA) class III or IV)

Breathlessness severity: Modified MRC dyspnoea scale grade 3 or 4 (i.e. stops for breath after walking about 100 yards or after a few minutes on level ground, or is too breathless to leave the house or is breathless when dressing) .

On optimal treatment of the underlying condition in the opinion of the identifying clinician (see section 1.12.3 of protocol for guidance)

Management of the underlying condition unchanged for the previous 1 week

Reversible causes of breathlessness optimally treated in the opinion of the identifying clinician[i]

Expected prognosis of ≥2 months

If female, must be (as documented in patient notes):

a) postmenopausal (no menses for 12 months without an alternative medical cause), or
b) surgically sterile (hysterectomy, bilateral salpingectomy or bilateral oophorectomy), or
c) using acceptable contraception [ii] (which must be continued for 7 days after the last dose of IMP)

Able to complete questionnaires and trial assessments

Able to provide written informed consent

1.9 Exclusion Criteria

1. Existing antidepressant use[iii], use of linezolid, or St John's wort

[i] According to the current appropriate society national guidance.
[ii] Acceptable contraception is defined as one of the following: combined (estrogen and progestogen containing) hormonal contraception associated with inhibition of ovulation (oral, intravaginal or transdermal); progestogen-only hormonal contraception associated with inhibition of ovulation (oral, injectable, implantable); intrauterine device (IUD); intrauterine hormone-releasing system (IUS); bilateral tubal occlusion; vasectomised partner; practising true abstinence (when this is in line with the preferred and usual lifestyle of the subject).
[iii] Previous antidepressant use is permitted provided there is a wash-out period of 14 days prior to randomisation.

Higginson IJ, *et al. Thorax* 2020; 75:176–179. doi: 10.1136/thoraxjnl-2019-213879

2. Known contraindication to mirtazapine[iv]
3. Hypersensitivity to the active substance or to any of the components of the mirtazapine or placebo (e.g. lactose intolerance)
4. Australia modified Karnofsky Performance Scale ≤40[v]
5. Pregnant or breast-feeding women [vi]
6. Patients with acute cardiac events within 3 months of randomisation (myocardial infarction, unstable angina pectoris, or significant cardiac conduction disturbance)
7. Patients with known hepatic impairment
8. Patients with known renal impairment
9. Patients with uncontrolled blood pressure
10. Patients with uncontrolled diabetes mellitus
11. Patients with uncontrolled seizures, epilepsy or organic brain syndrome
12. Patients with severe depression or suicidal thoughts
13. Patients with a history of psychotic illness (schizophrenia, bipolar disorder, mania or hypomania, or other psychotic disturbances)

Recruitment Process

1.10 Recruitment Setting

Participants will be recruited from approximately three trial sites with trial coordination and data collection performed by the Clinical Trials Research Unit (CTRU) in Leeds. Participants may be identified from within the trial sites, or referred to the trial sites by community services, hospices and various other settings (e.g. patient support groups, etc.) Trial sites will be required to have obtained management approval and undertake a site initiation meeting with the Sponsor, and CTRU prior to the start of recruitment into the trial. Screening and recruitment processes must not be initiated at site until approval to open to recruitment has been formally issued by the CTRU.

[iv] One class of contraindicated concomitant medications listed in the mirtazapine Summary of Product Characteristics (SPC) are monoamine oxidase inhibitors (MAO-Is). Where a patient has previously taken a MAO-I, they must not be treated with mirtazapine for 14 days from the last dose.
[v] i.e. in bed more than 50% of the time, due to association with short survival.

[vi] for women of childbearing potential (those not post-menopausal or surgically sterile) must be confirmed by a pregnancy test within 7 days prior to randomisation

Higginson IJ, *et al. Thorax* 2020; 75:176–179. doi: 10.1136/thoraxjnl-2019-213879

The trial aims to recruit 60 participants over a 12-month period.

1.11 Screening

Patients diagnosed with cancer, heart failure or lung disease (COPD or ILD) and who have significant breathlessness[vii] will be screened for trial entry. All participating trial sites will be required to complete monthly Screening Logs of all patients screened for entry into the trial who do not go on to be randomised. This information will be collected from trial sites on a regular basis. Documented reasons for ineligibility or declining participation will be closely monitored by the CTRU.

1.12 Informed Consent and Eligibility

The Principal Investigator (PI) will retain overall responsibility for the informed consent of participants at their site and must ensure that any person delegated responsibility to participate in the informed consent process is duly authorised, trained and competent to participate according to the ethically approved protocol, principles of Good Clinical Practice and Declaration of Helsinki 1996. Informed consent must be obtained by the PI, or another medically qualified member of the team authorised to consent by the PI on the BETTER-B delegation log, prior to the participant undergoing procedures that are specifically for the purposes of the trial and are not standard routine care at the participating site.

Assenting participants will be broadly assessed for eligibility during the screening process based on their medical history according to the inclusion and exclusion criteria.

The right of a patient to refuse participation without giving reasons will be respected. The participant must remain free to withdraw at any time from the trial without giving reasons and without prejudicing his/her further treatment, and will be provided with a contact point where he/she may obtain further information about the trial. Where a participant is required to re-consent, or new information is required to be provided to a participant, it is the responsibility of the PI to ensure this is done in a timely manner and according to any timelines requested by the CTRU.

As this is a feasibility trial, we will investigate reasons why patients decline to participate using a Feedback questionnaire: completion of this questionnaire is entirely optional. Patients who decline entry into the trial are provided with the questionnaire and an envelope in which they can seal their completed questionnaire before returning to the research team. The questionnaire is provided at the time the patient refuses participation; this may be when they

[vii] For the purposes of this protocol, “significant breathlessness” is defined as an **anticipated** score of grade 3 or grade 4 on the modified Medical Research Council (mMRC) dyspnoea scale.

Higginson IJ, *et al. Thorax* 2020; 75:176–179. doi: 10.1136/thoraxjnl-2019-213879

are first approached or after they have had time to consider the participant information sheet. There is also a planned qualitative sub-study which will involve patients being interviewed (see Appendix D). Patients who decline to participate in the trial will still be eligible for this sub-study. For those decliners who choose to complete the Feedback questionnaire, there is a section therein for them to indicate if they would be happy to be approached for this sub-study at a future time. For those who do decide to participate in the main trial, the trial consent form includes a section for them to indicate if they would be happy to be approached for this sub-study.

1.12.1 Initial Information and Initial Approach

Potential participants may be identified through a variety of methods: by staff at the recruiting site itself (e.g. through hospital clinic lists, searching of existing hospital databases, cancer Multi-Disciplinary Teams (MDT) meetings, etc.), by staff at Participants Identification Centres (PICs), and through the use of trial publicity in various settings (e.g. hospices, patient support groups, etc.).

The use of existing hospital databases of patients who have previously consented to be contacted about research may be used and initial contact with these patients will be in-line with what they had previously agreed with that site (e.g. initial contact by phone, or by letter, etc.). Potential participants identified through such databases may be contacted directly by the trial site's research team (if such contact has previously been agreed by the patient), or alternatively may be approached via an 'Invitation' Letter which will provide contact details of the recruiting site's research team. Brief trial information in the form of a Participant Summary Leaflet (PSL) will also be provided.

Potential participants identified at a PIC, who agree to receive further information about the trial, will be provided with the PSL and will be asked if their details may be passed on to the research team at the nearest recruiting site so they can be contacted. If the potential participant is interested in participating in the trial, they will also be provided with the contact details of the research team so they can themselves contact the recruiting site's research team directly if they so choose.

Trial publicity (posters and PSLs) will also be available in various NHS and non-NHS settings (non-NHS hospices, patient support groups, etc.) and these will include contact details of the nearest recruiting site's research team. Potential participants may directly contact a recruiting site's research team via the use of such publicity.

Once the potential participant has been contacted by, or have themselves contacted, the recruiting site's research team, an appointment will be made to meet with a member of the research team to discuss the trial further. At this visit, they will be provided with further verbal explanation of the trial, the Participant Information Sheet (PIS) and Informed Consent Form

Higginson IJ, *et al. Thorax* 2020; 75:176–179. doi: 10.1136/thoraxjnl-2019-213879

(ICF), which include detailed information about the rationale, design and personal implications of the trial.

1.12.2 Consent Process

Following initial information provision, participants will have as long as they need to consider participation in the trial (usually at least 24 hours) and will be given the opportunity to discuss the trial with their family and other healthcare professionals before they are asked whether they would be willing to take part in the trial.

Assenting potential participants will be invited to provide written informed consent. The PI or any other delegated medic who has received Good Clinical Practice (GCP) training and is authorised on the trial Delegation Log is permitted to take informed consent for trial participation.

Where the patient is able to provide fully informed consent but is unable to sign or otherwise mark the consent form, provision for completion of the consent form by a witness will be made. This should be a carer, friend/family member, or a local member of the clinical team who is independent of the research team.

A record of the consent process detailing the date of consent and all those present will be kept in the participant's medical notes. The original signed consent form(s) will be filed in the Investigator Site File, a copy will be given to the participant, a copy will be returned to the CTRU and another copy will be filed in the hospital notes (as per local practice).

Where valid informed consent is obtained from the participant and the participant subsequently becomes unable to provide ongoing informed consent by virtue of physical or mental incapacity, the consent previously given when capable remains legally valid. Participants who lose capacity after informed consent has been obtained will continue with protocol treatment, assessments and follow-up in consultation with the PI and participant's carer / family with the participant's best interests foremost in the decision-making process. Ongoing collection of safety and follow-up data will continue via the clinical care team for inclusion in the trial analysis in order to preserve the integrity of the trial's intention to treat analysis and fulfil regulatory requirements specifically for pharmacovigilance purposes. The PI will take responsibility for ensuring that all vulnerable subjects are protected and participate voluntarily in an environment free from coercion or undue influence.

1.12.3 Eligibility Process

The following assessments must be carried out **prior to randomisation** in order to establish eligibility (see section 0 above for full eligibility criteria):

Higginson IJ, *et al. Thorax* 2020; 75:176–179. doi: 10.1136/thoraxjnl-2019-213879

- Medical review (including medical history, assessment of management of the underlying disease and concomitant medication use)
- mMRC dyspnoea scale assessment
- AKPS assessment
- Pregnancy test for women of childbearing potential (those not post-menopausal or surgically sterile)

The assessment of whether the participant is receiving optimal treatment for their underlying disease is to be made by the identifying clinician and should be based on the following guidance:

- For COPD or ILD:[viii]
  - On optimal immunosuppression for Connective Tissue Disease (CTD) ILD
  - On pirfenidone for IPF if suitable [70, 71].
  - On oxygen if hypoxic at rest or on activity
  - On appropriate treatment for pulmonary hypertension, if applicable
  - Had pulmonary rehabilitation if appropriate.

- For heart failure:
  - Reached target dose (or be on maximally tolerated dose, or be intolerant) of an inhibitor of the renin-angiotensin system shown to improve prognosis;

  AND
  - Reached target dose (or be on maximally tolerated dose, or be intolerant) of a beta adrenoceptor antagonist shown to improve prognosis;

  AND
  - Reached target dose (or be on maximally tolerated dose, or be intolerant) of an aldosterone antagonist.
- For cancer: chemotherapy, radiotherapy or other anti-cancer treatment not currently appropriate or planned, as assessed at MDT meeting including oncologists, surgeons and relevant specialists, with review of radiological and histological data.

Informed consent must be obtained prior to undertaking any trial-specific procedures, including non-routine eligibility assessments. All eligibility assessments must be performed no more than 7 days prior to the participant being randomised and beginning trial treatment. Where more than 7 days have elapsed since the initial eligibility assessments, these must be

[viii] Based on NICE Idiopathic Pulmonary Fibrosis (IPF) / pirfenidone guidelines, British Thoracic Society ILD guidelines (includes CTD assoc ILD), American Thoracic Society (ATS) / European Respiratory Society (ERS) guideline ILD.

Higginson IJ, *et al. Thorax* 2020; 75:176–179. doi: 10.1136/thoraxjnl-2019-213879

repeated prior to randomisation and the participant beginning trial treatment; if these repeated assessments show ineligibility, the patient must not be randomised into the trial.

1.13 Randomisation

1.13.1 Timing of Randomisation

Informed written consent for entry into the trial must be obtained, and baseline assessments[ix] performed prior to randomisation. Following confirmation of written informed consent and eligibility, participants will be randomised into the trial by an authorised member of staff at the trial site. Randomisation will be performed centrally using the CTRU 9:00 – 17:0-00 (office hours)[x] randomisation system and should take place as soon as possible after consent is obtained and eligibility confirmed, and no more than 7 days prior to the start date of trial treatment.[xi]

1.13.2 Treatment Allocation

Participants will be randomised on a 1:1 basis to receive either mirtazapine or placebo and will be allocated a trial number and a unique kit-code to identify which container of trial drug (mirtazapine or placebo) will be dispensed. The participant's randomisation allocation will not be disclosed in order to maintain the blinding of the trial.

A computer-generated minimisation programme that incorporates a random element will be used to ensure that treatment groups are well balanced by:

- Disease (cancer vs non-cancer)
- HADS score (≥15 vs <15)
- Currently receiving opioids (yes vs no)

1.13.3 Randomisation Process

Randomisation should take place as soon as possible after consent is obtained and eligibility confirmed, and must be performed by an authorised member of the team at the site using the CTRU office hours telephone randomisation service (open 9:00 to 17:00 Monday to Friday, excluding public / bank holidays, the period between Christmas Eve and New Year, Thursday

[ix] It is important that baseline assessments are performed prior to randomisation, as the HADS score is used as a minimisation factor.
[x] Exceptions: public / bank holidays, the period between Christmas Eve and New Year, Thursday afternoon before Good Friday and all Tuesdays following a bank holiday except for Mayday and New Year's Day.
[xi] Where this is not possible, the eligibility assessments must be repeated so that they are no more than 7 days old at the time of starting treatment; if the repeated assessments show ineligibility, the patient must not be entered into the trial.

Higginson IJ, *et al. Thorax* 2020; 75:176–179. doi: 10.1136/thoraxjnl-2019-213879

afternoon before Good Friday and all Tuesdays following a bank holiday except for Mayday and New Year's Day).

The following information is required in order for the participant to be randomised. The person making the randomisation telephone call should have all details to hand:

- Name and code (assigned by the CTRU) of trial site
- Patient initials and date of birth
- Confirmation of eligibility
- Confirmation of written informed consent
- Minimisation factors (see section 1.13.2 above)

Direct line for office hours randomisation
0113 343 1486
Please ensure that you have completed the Initial Eligibility Checklist and Randomisation Case Report Forms (CRFs) before telephoning

At the end of this phone call a unique BETTER-B trial participant identifier will be assigned but the participant's randomisation allocation will not be disclosed in order to maintain the blinding of the trial. Instead, a unique kit-code will be provided which identifies a container of capsules that need to be dispensed by pharmacy.

1.13.4 Post-Randomisation Actions

At the end of the randomising phone call, the trial participant identifier and kit-code number must be added to the Randomisation Case Report Form (CRF) and all participant details must be added to the main Participant ID Log.

Two Confirmation of Randomisation notifications, detailing the participant details and the kit-code number they have been allocated will be sent to site: one to the nominated contact in the local research team and another to pharmacy. These should be filed in the Investigator Site File and Pharmacy Site File, respectively. The kit-code provided will inform pharmacy which container of capsules needs to be dispensed to the participant. These notifications are generated and sent from the CTRU. In the event of a system failure, the kit-code number may need to be provided to the pharmacy directly by the member of site staff randomising the participant (this information will be provided as part of the randomising phone-call).

Higginson IJ, *et al. Thorax* 2020; 75:176–179. doi: 10.1136/thoraxjnl-2019-213879

Trial Medicinal Product Management

Please refer to the BETTER-B Pharmacy and Investigational Medicinal Product (IMP) Study Site Operating Procedure (SSOP) for full details of the trial IMP management requirements. Within the trial the following are classed as IMPs:

Mirtazapine

- Composition: one capsule contains 15mg of mirtazapine.
- Supplied by Medreich Plc.

Placebo

- Composition: gelatin capsule shell containing lactose.
- Manufactured by Guy's and St Thomas' NHS Foundation Trust's (GSTFT) Pharmacy Production Unit.

For handling guidance of both mirtazapine and placebo, please refer to the latest Summary of Product Characteristics (SPC) for mirtazapine (as supplied by Medreich Plc; PL number 21880/0053).

1.14 GSTFT Manufacture, packaging and Labelling

The Pharmacy Production Unit at GSTFT will act as the trial's Central Pharmacy and holds a Manufacturer's Authorisation for IMPs.

The trial IMP placebo will be manufactured by the trial Central Pharmacy. The trial IMP mirtazapine will be sourced by the Central Pharmacy where the capsules will be over-encapsulated in such a way that they are identical to the placebo capsules in order to maintain the blind of the trial.

The Central Pharmacy will also package up the trial IMPs (each container will hold 42 capsules or either placebo or over-encapsulated mirtazapine) and label the containers. In order to maintain the blinding of the trial the capsules and containers will be identical and labelled with the same study-specific label in accordance with Directive 2001/20/EC and the Medicines for Human Use (Clinical Trials) Regulations 2004 (as amended).

Containers will be identified only by a unique kit-code assigned at random. Management of kit-codes on the kit logistics application will be conducted by the CTRU Trial Statistician in addition to maintaining the back-up kit-code lists for each site. The CTRU Trial Statistician will be responsible for maintaining this list, which will be securely password-protected when treatment information is contained within the list.

Higginson IJ, *et al. Thorax* 2020; 75:176–179. doi: 10.1136/thoraxjnl-2019-213879

1.15 Supply, Distribution and Storage

Trial IMPs (mirtazapine and placebo) will be provided to sites free of charge for use in this clinical trial. Blinded supplies will be sent to trial sites from Central Pharmacy (see above) in pre-labelled containers which will be identifiable by a kit-code printed on the label. In addition to the trial IMP containers, sites will receive sealed Code-Break Envelopes to allow emergency unblindings where necessary. Each envelope will be linked to a specific container of trial IMP capsules using the unique kit-code system. See section 1.19 below for further details on emergency unblinding.

Once received from the trial Central Pharmacy, all trial IMP stock and Code-Break Envelopes must be documented as received in accordance with the BETTER-B Pharmacy and IMP SSOP provided within the BETTER-B Pharmacy Site File.

All trial IMP containers must be stored in a secure ring-fenced location within the site pharmacy. There are no special storage requirements in terms of temperature management.

The supply of trial IMPs (mirtazapine and placebo) must not be used for any purpose other than that outlined in this protocol and should be clearly ring-fenced from standard hospital stock.

1.16 Dispensing

In order to maintain the blinding of the trial the site pharmacist will not be told the participant's treatment allocation. Blinded containers of capsules, identifiable only by a unique kit-code are received at site pharmacies from the Central Pharmacy and will be stored in a ring-fenced section of the site pharmacy until dispensing. To ensure that the correct treatment is dispensed to the participant the relevant site pharmacist will be told which container to dispense to each participant using this kit-code numbering system.

The relevant site pharmacist will be notified by the CTRU of all participants randomised at that site; each Confirmation of Randomisation notification will detail the participant trial ID number, date of birth and initials and also the kit-code assigned to that participant which will identify which container of capsules should be given to the participant. The member of the local research team randomising the participant will also have been told which kit-code should be dispensed to the participant whilst making the randomisation telephone call, and will also receive a Confirmation of Randomisation Fax detailing the kit-code.

The participant's trial identifier must be added to the label of each trial IMP container by the pharmacist (or authorised delegate) at the time of dispensing, and the Code-Break Envelope assigned to that kit-code annotated with the participant details and then securely stored in pharmacy.

Dispensing must only be performed by an authorised member of site staff as delegated on the trial Pharmacy Authorised Personnel Log. Once randomised, at baseline the participant will receive one trial IMP container, containing 42 capsules, of either mirtazapine or placebo, identifiable only by the unique kit-code on the outer container label. All dispensed trial IMP must be recorded on the trial Accountability and Dispensing Log in accordance with the BETTER-B Pharmacy and IMP SSOP.

Each container of trial IMP capsules, identifiable only by the unique kit-code, will have a corresponding Code-Break Envelope. Each time a container of capsules is dispensed, the participant identifiers must be added to the trial IMP container label and also to the corresponding Code-Break Envelope. This Code-Break Envelope will then be held securely within the site pharmacy (see section 1.19 for access required in the event of unblinding).

1.17 Reconciliation

All trial IMP stock received by site pharmacies from Central Pharmacy, dispensed to trial participants (and any returned unused doses from participants) must be recorded on the BETTER-B Accountability and Dispensing Logs. These completed logs will be reviewed by Sponsor at monitoring visits. Trial IMP stock (dispensed and returned, or un-dispensed) may only be destroyed by trial site pharmacies once full reconciliation has been performed by Sponsor and formal permission for destruction issued.

Code-Break Envelopes for all trial IMP containers (whether or not dispensed) will be returned to CTRU at the end of trial for destruction.

BETTER-B Treatment

1.18 Treatment Details

The local Investigator, the site pharmacist, other members of the site staff involved with the trial, and the participants themselves, will remain blinded to the treatment allocation (except where emergency unblinding is necessitated).

1.18.1 Treatment Regimen

Participants will be randomised to receive either mirtazapine or placebo for 28 days. Participants will be dispensed 42 capsules (15mg per tablet for mirtazapine) at baseline. Participants should take their capsules in the evening.

Higginson IJ, *et al. Thorax* 2020; 75:176–179. doi: 10.1136/thoraxjnl-2019-213879

For participants randomised to receive mirtazapine, the daily dose will be 15mg (one capsule) for the first 14 days; participants will be assessed for possible dose escalation at the trial assessment visit for day 14 and if appropriate, their daily dose will be escalated to 30mg (two capsules) on days 15 through to 28; see section 0 below for further details.[xii] Where dose escalation is not appropriate, the participant will continue to take a daily dose of 15mg (one capsule) on days 15 through to 28.

For participants randomised to receive placebo the daily dose will be 1 capsule for the first 14 days; participants will be assessed for possible dose escalation on day 14 and if appropriate, their daily dose will be escalated to 2 capsules on days 15 through to 28; see section 0 below for further details.[xiii] Where dose escalation is not appropriate, the participant will continue to take a daily dose of 1 capsule on days 15 through to 28.

1.18.2 Treatment Compliance

In order to assess participant compliance with the trial treatment, at the assessment phone-calls (day 7 and day 21) and visits (day 14 and day 28) the research team will ask the participant if they have had any delayed, missed or modified doses. This information will be recorded on the appropriate Assessment CRF. Any unused capsules should be collected from the participants by the research team at the last assessment visit (day 28)[xiv] and returned to pharmacy for drug reconciliation then destruction (see section 1.17 above for further details).

Participants will be given a medication diary to complete in order to aid in the monitoring of treatment compliance. This diary will be given to participants at baseline and they will be asked to complete this every day and to bring it along to all trial visits (days 14 and 28) and have it available during trial calls (days 7, 21 and follow-up call at 7 days after ending trial treatment).

1.18.3 Concomitant Medications / Interactions

For management of concomitant therapies, please refer to the latest mirtazapine Summary of Product Characteristics (produced by Medreich Plc; PL 21880/0053).

Use of MAO inhibitors, linezolid, other antidepressant medication, and St John’s wort are prohibited for the participant for the duration of the trial. Furthermore, MAO inhibitors must not be used until there has been a 14 day washout period from the last dose of IMP.

[xii] Where dose escalation is deemed appropriate for a participant, and the Day 14 trial visit occurs before the 14th day of treatment, the participant must be instructed to begin taking two capsules per day only from day 15 onwards. Where dose escalation is deemed appropriate for a participant, and the Day 14 trial visit occurs after the 14th day of treatment, the participant must be instructed to begin taking two capsules per day from that point forward until the end of their trial treatment (i.e. day 28).
[xiii] See footnote xii above.
[xiv] If the Day 28 visit occurs before the 28th day of trial treatment, all capsules should be left with the participant at that visit, and another visit arranged for collection of unused capsules after the participant has completed 28 days of treatment.

Higginson IJ, *et al. Thorax* 2020; 75:176–179. doi: 10.1136/thoraxjnl-2019-213879

Caution is advised (particularly in relation to dose escalation) when used with inhibitors or inducers of CYP3A4, benzodiazepines, alcohol and warfarin.

1.18.4 Most frequent anticipated toxicities

The most frequent anticipated toxicities of mirtazapine are as follows:

- Increased appetite
- Weight gain
- Somnolence
- Sedation
- Headache
- Dry mouth
- Lethargy
- Dizziness
- Tremor
- Nausea
- Diarrhoea
- Vomiting
- Exanthema
- Arthralgia
- Myalgia
- Back pain
- Orthostatic hypotension
- Oedema peripheral
- Fatigue
- Abnormal dreams
- Confusion
- Anxiety
- Insomnia

1.19 Emergency Unblinding

Whilst the safety of participants in the trial must always take priority, maintenance of blinding is crucial to the integrity of the trial. Investigators should only break the blind when information about the participant‘s trial treatment is clearly necessary for the appropriate medical management of the participant and where stopping the blinded medication is not sufficient.

Unblinding may be requested on the grounds of safety by the Chief Investigator (CI), local PI or treating physician. It is anticipated that requests for unblinding will most likely originate from a participant, carer (or friend / family member) or personal physician (e.g. GP) at the time of an adverse event or planned change in non-trial related drug therapy. Requests for unblinding will first be handled by the local PI or delegate who will explore the reason for the request and evaluate the importance of knowledge of treatment assignment for participant safety. In the event of a Serious Adverse Event (SAE), all participants should be treated as though they are receiving the active medication.

Should an alternative to unblinding not be identified, and if unblinding is required to optimise medical management of the participant, investigators should follow the emergency unblinding process.

Emergency unblinding is provided by the CTRU during Office Hours and the participating site pharmacy at all other times, thereby covering each 24-hour period. It

Higginson IJ, *et al. Thorax* 2020; 75:176–179. doi: 10.1136/thoraxjnl-2019-213879

is encouraged that requests for Emergency Unblinding should be made directly with CTRU wherever possible.

The following information will be needed to perform an emergency unblinding:

- Participant details, including trial ID number, initials and date of birth
- Name of trial research site and site code
- Name of person making the request for a code-break
- Reason for requesting a code-break
- Confirmation of whether the PI authorised the request

1.19.1 Emergency Unblinding during Office Hours

The emergency unblinding process will be undertaken by telephoning the CTRU during Office Hours, 9.00 to 17.00 Monday to Friday. Exceptions: public / bank holidays, the period between Christmas Eve and New Year, Thursday afternoon before Good Friday and all Tuesdays following a bank holiday except for Mayday and New Year's Day.

Direct line for CTRU emergency unblinding: 0113 343 1486

Following the emergency unblinding of a participant, CTRU will send a notification to the requester, the local PI and the Sponsor. The details of the emergency unblinding should be recorded on the BETTER-B Unblinding Log provided by CTRU.

1.19.2 Emergency Unblinding outside of Office Hours

Outside of Office Hours, or where the Investigator or treating physician is unable to contact CTRU, emergency unblinding must be performed by the local pharmacy department. The responsible pharmacist on duty will complete the Unblind Request CRF, retrieve the code-break information (Code-Break Envelopes for unblindings will be provided to pharmacy at the time of IMP delivery and each envelope will be linked to a specific container of capsules using a unique kit-code) and reveal the treatment allocation to the person requesting the unblind. The pharmacist must send the completed Unblind Request CRF to the CTRU within 24 hours of the unblinding request (please see section 0 for details of acceptable methods of transfer).

Higginson IJ, *et al. Thorax* 2020; 75:176–179. doi: 10.1136/thoraxjnl-2019-213879

All Code-Break Envelopes will be returned to CTRU by the site pharmacy department at the end of trial. Code-Break Envelopes must not be opened for participants when they have completed trial therapy.

1.19.3 Treatment of Participants following Emergency Unblinding

Following an emergency unblinding the participant should be treated according to the treating clinician's assessment.

1.20 Withdrawal of Treatment

In line with usual clinical care, cessation or alteration of regimens at any time will be at the discretion of attending clinicians or the participants themselves. All participants withdrawn from treatment or prescribed alternative treatment will still attend for follow-up assessments unless unwilling to do so and CRFs will continue to be completed.

The PI, or delegate should make every effort to ensure that the specific wishes of any participant who wishes to withdraw consent for further involvement in the trial are defined and documented using the Withdrawal CRF in order that the correct processes are followed by the CTRU and site following the withdrawal of consent.

It should be made clear to any participant specifically withdrawing consent for further data collection that data pertaining to safety will continue to be collected for regulatory reporting purposes and will be included in any safety analysis. In addition it is suggested that the participant is made aware of the fact that if any significant new information becomes available in regard to the treatment they have received in the trial it may be necessary to contact them in the future.

Assessments and Data Collection

1.1 Schedule of Events

The timings of interventions and assessments required for the BETTER-B Feasibility trial are summarised in Table 1.

Table 1: Schedule of Events

Abbreviations: mMRC (modified Medical Research Council), AKPS (Australia-modified Karnofsky Performance Status), Tx (treatment), NRS (Numerical Rating Scale), CRQ (Chronic Respiratory Questionnaire), SPPB (Short Physical Performance Battery), GSES (Generalized Self-Efficiency Scale), IPOS (Integrated Palliative care Outcome Scale), CSRI (Client Services Receipt Inventory).

TIMEPOINT	Eligibility (≤ 7 calendar days prior to starting treatment)	Baseline (≤ 7 calendar days prior to starting treatment)	Day 7 (+/- 1 working day)	Day 14 (+/- 1 working day)	Day 21 (+/- 1 working day)	Day 28 (-1 working day)	7 days post treatment end (+1 working day)
**ASSESSMENTS**	Face-to-face	Face-to-face	Phone-call	Face-to-face	Phone-call	Face-to-face	Phone-call
Demographic data and full medical review [disease, prognosis, optimal Tx, concomitant medications, contraindications, cardiac history, symptoms]	X[1]	X[2]					
Pregnancy test (for women of childbearing potential)	X[1]					X	
Randomisation and dispensing		X					
mMRC dyspnoea scale (participant-reported)	X[1]	X[2]		X		X	
CRQ (participant-reported)		X		X		X	
GSES (participant-reported)		X				X	
IPOS (participant-reported)		X		X		X	
HADS (participant-reported)		X		X		X	
CSRI (participant-reported)		X				X	
EQ-5D-5L (participant-reported)		X				X	
NRS (participant-reported)		X	X	X	X	X	
AKPS	X[1]	X[2]		X		X	
SPPB		X				X	
Vital signs (blood pressure and blood oxygen level)				X		X	
Toxicity assessment		X	X	X	X	X	X
Opioid medication assessment		X	X	X	X	X	
Mirtazapine compliance assessment (and modifications)			X	X	X	X[3]	X[3]
Suitability for dose escalation				X		X	
Blinding assessment						X	
Collection of unused medication						X[3]	

1. Eligibility assessments must be no more than 7 days before starting treatment. Where more than 7 days elapse from the initial eligibility assessments, they must be repeated before randomisation and starting treatment.
2. Eligibility assessments may be used for baseline so long as they are no more than 7 days old at the time starting treatment.
3. If the participant has not completed 28 days of trial treatment at the time of the Day 28 trial visit, trial IMP compliance will be assessed at the follow-up call (7days after completing trial treatment) and an unused trial IMP collected in.
4. If the participant has not completed 28 days of trial treatment at the time of the Day 28 trial visit, another visit must be arranged in order to collect any unused trial IMP.

Higginson IJ, *et al. Thorax* 2020; 75:176–179. doi: 10.1136/thoraxjnl-2019-213879

1.2 Screening Data

All patients who have significant breathlessness[xv] but do not go on to be randomised must be included on the monthly Screening Log[xvi]. Anonymised information for these patients will be collected including:

- Disease area diagnosis (e.g. cancer, heart or liver disease)
- Identification setting (community services, outpatient clinic, inpatient clinic)
- Method of initial approach
- Date screened
- Approached / Not approached for the trial
- Reason for non-randomisation:
  - not eligible for trial participation, or
  - eligible but declined and reason for this (where appropriate), or
  - other reason for non-randomisation

This information will be collected from trial sites on a monthly basis. Documented reasons for ineligibility or declining participation will be closely monitored by the CTRU. Screening data forms a crucial endpoint of this feasibility study therefore it is essential that this information is completed and returned to CTRU as outlined.

1.3 Eligibility Assessments

The following assessments need to be performed in order to assess eligibility (see section 0 above for full eligibility criteria):

- Medical review (including medical history, assessment of management of the underlying disease and concomitant medication use)
- Modified Medical Research Council (mMRC) dyspnoea scale assessment
- Australia-modified Karnofsky Performance Scale (AKPS) ≤40
- Pregnancy test for women of childbearing potential (those not post-menopausal or surgically sterile)

[xv] For the purposes of this protocol, "significant breathlessness" is defined as an **anticipated** score of grade 3 or grade 4 on the modified Medical Research Council (mMRC) dyspnoea scale.
[xvi] If a participant's screening process spans more than one month, their details should only be included on the Screening Log for the month that the screening outcome is final.

Eligibility assessments must be no more than 7 days old at the time of starting treatment; if more than 7 calendar days elapse from the date of the eligibility assessments and the participant has not commenced trial treatment, all eligibility assessments must be repeated.

For patients who do not go on to be randomised, details should be added to the Screening Log (see section 1.11).

1.4 Baseline Assessments and Data Collection

Following written informed consent and prior to randomisation[xvii], the participant will be assessed by a member of the research team and the following baseline assessments will be carried out. This visit may be conducted either at the trial site, or, if the participant prefers, at the participant's home / agreed convenient location (e.g. care home, etc.)[xviii].

Assessments to be performed by the research team:

- Medical review
- Australia-modified Karnofsky Performance Scale (AKPS)[xix]
- Short Physical Performance Battery (SPPB)
- Numerical Rating Scale (NRS) – on average and "at worst" in the last 24 hours

A number of participant-reported questionnaires will also be completed. These may be completed by the participants themselves, or, if preferred, by a member of the research team on behalf of the participant. Where this is the case, separate booklets are provided for staff to complete, along with a number of laminated "prompt sheets" to be given to the participant to facilitate this process:

- Modified Medical Research Council (mMRC) dyspnoea scale[xx]
- Chronic Respiratory Questionnaire (CRQ)
- Integrated Palliative care Outcome Scale (IPOS)

[xvii] The baseline HADS score will be used at randomisation as a minimisation factor. Ideally, randomisation and day 1 of trial treatment should occur on the same day.
[xviii] Where research team members visit a participant's home, they should follow their local "lone worker" policy to minimise any risks to their personal safety.
[xix] The AKPS assessment performed for eligibility may be used (this must not be more than 7 days prior to starting treatment).
[xx] The mMRC dyspnoea scale assessment performed for eligibility may be used (this must not be more than 7 days prior to starting treatment).

- Hospital Anxiety and Depression Scale (HADS)
- EQ-5D-5L
- Generalized Self-Efficacy Scale (GSES)
- Client Services Receipt Inventory (CSRI) [72]

1.5 Trial Treatment Assessments and Data Collection

1.5.1 Day 7 Assessment Phone Call

On day 7[xxi] of trial treatment, the research team will contact the participant by phone to perform the following assessments:

- Numerical Rating Scale (NRS) – on average and "at worst" over the last 24 hours
- Toxicity assessment: collection of any adverse events or reactions which may have occurred since the baseline assessment[xxii]
- Opioid medication assessment: collection of information of any opioids taken by the participant since the baseline assessment
- Treatment compliance (and any modifications) assessment since the baseline assessment (mirtazapine or placebo)

1.5.2 Day 14 Assessment Visit and potential dose escalation

On day 14[xxiii] of trial treatment, the participant will be seen in person by a member of the research team and the following assessments will be carried out. This visit may be conducted either at the recruiting trial site, or, if the participant prefers, at another location of their choice (e.g. the participant's home, care home, etc.)[xxiv].

Assessments to be performed by the research team:

- Australia-modified Karnofsky Performance Scale (AKPS)
- Numerical Rating Scale (NRS) – on average and "at worst" over the last 24 hours

[xxi] Where this phone-call cannot take place on day 7 of treatment, it should be no more than 1 working day either side.
[xxii] Where this toxicity assessment raises any safety concerns, the assessing research team member may request that the participant is assessed by a medically qualified member of the team.
[xxiii] Where this visit cannot take place on day 14 of treatment, it should be no more than 1 working day either side.
[xxiv] Where research team members visit a participant's home, they should follow their local "lone worker" policy to minimise any risks to their personal safety.

- Toxicity assessment: collection of any adverse events or reactions which may have occurred since the Day 7 assessment call[xxv]
- Opioid medication assessment: collection of information of any opioids taken by the participant since the Day 7 assessment call
- Treatment compliance assessment (and any modifications) since the Day 7 assessment call (mirtazapine or placebo)
- Assessment of appropriateness to dose escalate (see section 0 below)
- Medical review including vital signs (blood pressure and blood oxygen level)

A number of participant-reported questionnaires will also be completed. These may be completed by the participants themselves, or, if preferred, by a member of the research team on behalf of the participant. Where this is the case, separate booklets are provided for staff to complete, along with a number of laminated "prompt sheets" to be given to the participant to facilitate this process.

- Modified Medical Research Council (mMRC) dyspnoea scale
- Chronic Respiratory Questionnaire (CRQ)
- Integrated Palliative care Outcome Scale (IPOS)
- Hospital Anxiety and Depression Scale (HADS)

Dose Escalation

All BETTER-B participants will be assessed for dose escalation (to two capsules of mirtazapine (30mg total dose) or placebo daily) at their day 14 assessment visit. The assessment of suitability for dose escalation will be based on the participant's NRS score ("at worst" over last 24 hours), and clinical review. Participants will be eligible for dose escalation where their NRS score has not improved by at least 1 point since baseline. If participants have experienced toxicity since baseline, they will have a clinical review prior to being assessed as eligible for dose escalation.

For participants for whom it is determined that dose escalation is appropriate at the day 14 assessment visit, they should be instructed by the research team member to begin taking an additional capsule every day from day 15 onwards[xxvi] (so 2 capsules daily to be taken from day 15 through to day 28).

[xxv] Where this toxicity assessment raises any safety concerns, the assessing research team member may request that the participant is assessed by a medically qualified member of the team.
[xxvi] Or from the day of the trial assessment visit, where this occurs after day 14 of trial treatment.

Higginson IJ, *et al. Thorax* 2020; 75:176–179. doi: 10.1136/thoraxjnl-2019-213879

For participants for whom it is determined dose escalation is not appropriate at the day 14 assessment visit, they should be instructed to continue to take one capsule daily.

1.5.3 Day 21 Assessment Phone Call

On day 21[xxvii] of trial treatment, the research team will contact the participant by phone to perform the following assessments:

- Numerical Rating Scale (NRS) – on average and "at worst" over the last 24 hours
- Toxicity assessment: collection of any adverse events or reactions which may have occurred since the Day 14 assessment visit[xxviii]
- Opioid medication assessment since the Day 14 assessment visit
- Treatment compliance assessment (and any modifications) since the Day 14 assessment visit (mirtazapine or placebo)

1.5.4 Day 28 Assessment Visit

On day 28[xxix] of trial treatment, the participant will be seen in person by a member of the research team and the following assessments will be carried out. This visit may be conducted either at the recruiting trial site, or, if the participant prefers, at another location of their choice (e.g. the participant's home, care home, etc.)[xxx].

Assessments to be performed by the research team:

- Australia-modified Karnofsky Performance Scale (AKPS)
- Numerical Rating Scale (NRS) – on average and "at worst" over the last 24 hours
- Short Physical Performance Battery (SPPB)
- Toxicity assessment: collection of any adverse events or reactions which may have occurred since the Day 21 assessment call[xxxi]

[xxvii] Where this phone-call cannot take place on day 21 of treatment, it should be no more than 1 working day either side.

[xxviii] Where this toxicity assessment raises any safety concerns, the assessing research team member may request that the participant is assessed by a medically qualified member of the team.

[xxix] Where this visit cannot take place on day 28 of treatment, it **must not** be earlier than day 28 of treatment, and should not be more than 1 working day later.

[xxx] Where research team members visit a participant's home, they should follow their local "lone worker" policy to minimise any risks to their personal safety.

[xxxi] Where this toxicity assessment raises any safety concerns, the assessing research team member may request that the participant is assessed by a medically qualified member of the team.

Higginson IJ, *et al. Thorax* 2020; 75:176–179. doi: 10.1136/thoraxjnl-2019-213879

- Opioid medication assessment: collection of information (drug name and dose) of any opioids taken by the participant since the Day 21 assessment call
- Treatment compliance assessment (and any modifications) since the Day 21 assessment call (mirtazapine or placebo)
- Assessment of potential to dose escalate
- Medical review including vital signs (blood pressure and blood oxygen level)
- Pregnancy test for women of childbearing potential (those not post-menopausal or surgically sterile)

A number of participant-reported questionnaires will also be completed. These may be completed by the participants themselves, or, if preferred, by a member of the research team on behalf of the participant. Where this is the case, separate booklets are provided for staff to complete, along with a number of laminated "prompt sheets" to be given to the participant to facilitate this process.

- Modified Medical Research Council (mMRC) dyspnoea scale
- Chronic Respiratory Questionnaire (CRQ)
- Integrated Palliative care Outcome Scale (IPOS)
- Hospital Anxiety and Depression Scale (HADS)
- EQ-5D-5L
- Generalized Self-Efficacy Scale (GSES)
- Client Services Receipt Inventory (CSRI)
- Blinding Assessment

1.6 Follow-up Assessment and Data Collection

Participants will be followed-up 7 days[xxxii] after the end of trial treatment. The research team will contact the participant by phone to perform the following assessments:

- Toxicity assessment: collection of any adverse events or reactions which may have occurred since the participant stopped trial treatment

[xxxii] Where this phone-call cannot take place exactly 7 days after the end of trial treatment, it **must not** be earlier than 7days after completing treatment, and should be no more than 1 working day later.

Higginson IJ, *et al. Thorax* 2020; 75:176–179. doi: 10.1136/thoraxjnl-2019-213879

1.7 End of Trial Treatment

Participants should continue on trial treatment for 28 days, however if a participant discontinues trial treatment for any reason before that time, an End of Trial Treatment CRF must be completed and sent to the CTRU **within 7 days** of the research team becoming aware of this (please see section 0for details of acceptable methods of transfer).

1.8 Adverse Events and Serious Adverse Events

All Adverse Events (AEs) or Adverse Reactions (ARs) occurring in the trial will be collected on the weekly Trial Treatment Assessment CRFs and on the Follow-up Assessment CRF. These should be reported via the standard data management routes to the CTRU and not expedited.

For all Serious Adverse Events (SAEs) occurring in the trial, a SAE Report CRF must be completed and sent to the CTRU **within 24 hours** of the site becoming aware of the event (see pharmacovigilance section 0 and section 0 for details of acceptable methods of transfer).

For all Serious Adverse Reactions (SARs), a SAR Report CRF must be completed and sent to the CTRU **within 24 hours** of the site becoming aware of the event (see pharmacovigilance section 0 and section 0 for details of acceptable methods of transfer).

1.9 Pregnancies

All pregnancies and suspected pregnancies (in a trial participant or their partner) occurring from the date of randomisation to 7 days following permanent cessation of trial treatment must be reported to the CTRU by completing the Notification of Pregnancy CRF which must be sent to the CTRU **within 7 days** of the site becoming aware of the pregnancy (please see section 0 for details of acceptable methods of transfer).

The CTRU will report all pregnancies occurring during trial treatment to the Sponsor along with any follow-up information.

1.10 Deaths

All deaths occurring from the date of randomisation to 7 days after the participant has completed trial treatment must be recorded on the Notification of Death CRF and sent to the CTRU **within 7 days** of the site becoming aware of the death (please see section 0 for details of acceptable methods of transfer).

At the end of the trial, sites will be contacted to provide data on any subsequent deaths and survival data.

Higginson IJ, *et al. Thorax* 2020; 75:176–179. doi: 10.1136/thoraxjnl-2019-213879

1.11 Important Medical Events (IMEs)

Events that may not be immediately life-threatening or result in death or hospitalisation, but which may jeopardise the patient or require intervention to prevent one of the outcomes listed in the definition of a Serious Adverse Event (see section 1.16 below), should also be considered serious and should be expedited to the CTRU **within 24 hours** of the site becoming aware.

1.12 Protocol Deviations and Violations

The CTRU undertake to adopt all reasonable measures to record data in accordance with the protocol. Under practical working conditions, however, some minor variations may occur due to circumstances beyond the control of the CTRU. All such deviations or violations will be documented in the study records, together with the reason for their occurrence; where appropriate, deviations or violations will be detailed in the published report. We will analyse the reasons for deviations or violations and report on whether and how these might be avoided in a future large-scale trial.

1.13 End of Trial Definition

The end of trial is defined as the date of the collection of the last participant's last data item, i.e. the last participant's Follow-Up trial phone-call assessment, which will be no earlier than 7 days after the last participant has completed trial treatment.

1.14 Trial Data and Documentation held at sites

Participating sites must maintain essential trial documentation in an Investigator Site File and a Pharmacy Site File, which will be provided by the CTRU. It is the responsibility of the site staff to ensure these files are properly maintained during the trial and archived according to Sponsor requirements at the end of the trial (see section 0 on archiving).

1.15 Case Report Forms (CRFs)

Data will be recorded by site research staff on trial-specific paper CRFs which will be provided by CTRU in the form of an electronic booklet. The originals will be submitted by post to the BETTER-B trial team at CTRU within two weeks of the data being collected, and photocopies

of the completed CRFs will be held at site. A number of CRFs require expedited reporting to the CTRU:

- Within 24 hours of the site research team becoming aware: SAE and SAR CRFs, and notification of any IMEs
- Within 7 days of the research team becoming aware: Death, Notification of Pregnancy and End of Trial CRFs

Only the participant's trial number, date of birth and initials will be added to the CRFs – **site staff are responsible for ensuring the CRFs returned to CTRU do not contain any other personal identifiable data** (with the exception of the participant's NHS number which will be recorded at baseline). Following receipt of the completed CRFs, the CTRU will contact sites on a regular basis to resolve any missing or discrepant data.

It is the responsibility of the site to ensure all photocopies of the completed CRFs are appropriately maintained at site during the trial (including any amendments) and archived according to Sponsor requirements at the end of the trial (see section 0 on archiving).

Pharmacovigilance

1.16 General Definitions

The Medicines for Human Use (Clinical Trials) Regulations 2004 and Amended Regulations 2006 gives the following definitions:

Adverse Event (AE): any untoward medical occurrence in a subject to whom a medicinal product has been administered including occurrences which are not necessarily caused by or related to that product.

An AE can therefore be any unfavourable and unintended sign (Including an abnormal laboratory finding, for example), symptom or disease temporarily associated with the use of a medicinal product, whether or not considered to be related to the medicinal product.

Adverse Reaction (AR): any untoward and unintended response in a subject to an IMP which is related to any dose administered to that subject.

This definition implies a reasonable possibility of a causal relationship between the event and the IMP which is supported by facts, evidence or arguments to suggest a causal relationship. This definition includes medication errors and uses outside what is foreseen in the protocol (i.e. if an AR occurs as a result of a medication error), including misuse and abuse of the product.

Higginson IJ, *et al. Thorax* 2020; 75:176–179. doi: 10.1136/thoraxjnl-2019-213879

Serious Adverse Event (SAE): any adverse event that:

- Results in death;
- Is life-threatening;
- Required hospitalisation or prolongation of existing hospitalisation;
- Results in persistent or significant disability or incapacity;
- Consists of a congenital anomaly or birth defect.

These characteristics/consequences have to be considered at the time of the event. For example, regarding a life-threatening event, this refers to an event in which the subject was at risk of death at the time of event; it does not refer to an event which hypothetically might have caused death if it were more severe. Medical and scientific judgement must be exercised in deciding whether an event is ’serious’ in accordance with these criteria.

Serious Adverse Reaction (SAR): reference is made to the criterion of ‘Seriousness’ above in relation to SAE. Where an SAE is deemed to have been related to an IMP used within the trial, the event is termed as a SAR. (Any suspected transmission via a medicinal product of an infectious agent is also considered a serious adverse reaction.)

Suspected Unexpected Serious Adverse Reaction (SUSAR): an adverse reaction, the nature and severity of which is not consistent with the pharmacovigilance reference copy of the mirtazapine SPC (Medreich Plc; PL number 21880/0053).

The term ‘severity’ is used here to describe the intensity of a specific event. This has to be distinguished from the term ‘serious’. Reports which add significant information on the specificity, increase of occurrence, or severity of a known, already documented serious adverse reaction constitute unexpected events.

Important Medical Events (IME) & Pregnancy

Events that may not be immediately life-threatening or result in death or hospitalisation but may jeopardise the participant or may require intervention to prevent one of the other outcomes listed in the definitions above should also be considered serious.

Although not a serious adverse event, any unplanned pregnancy will also be reported to the CTRU in an expedited manner (i.e. within 7 days of the site becoming aware).

Death as a result of disease progression are not considered to be SAEs and should be reported in the normal way, on the appropriate CRF.

Higginson IJ, *et al. Thorax* 2020; 75:176–179. doi: 10.1136/thoraxjnl-2019-213879

1.17 BETTER-B Operational Definitions

Adverse events will be collected for all participants and will be evaluated for intensity and causal relationship with the trial medication or other factors according to the National Cancer Institute (NCI) CTCAE V4.0 (NCI-CTCAE). A copy is provided in the BETTER-B Investigator Site File and may also be obtained at:

http://ctep.cancer.gov/protocolDevelopment/electronic_applications/ctc.htm

Published date: May 28, 2009

1.17.1 Adverse Events (AEs) / Adverse Reactions (ARs) and Serious Adverse Events (SAEs) / Serious Adverse Reactions (SARs)

For general definitions of AEs, ARs, SAEs and SARs, please see section 1.16 above.

As this is a blinded trial, all AEs and SAEs should be assessed for causal relationship assuming that the participant has been receiving mirtazapine.

Routinely breaking the blind could compromise the integrity of the trial. For this reason blind-breaking will only take place where information about the participant's trial treatment is clearly necessary for the appropriate medical management of the participant. In all cases the Investigator would be expected to evaluate the causality of AEs or SAEs as though the participant was receiving the active medication.

When determining whether a SAE or SAR is expected or not, please refer to the pharmacovigilance reference copy of the mirtazapine Summary of Product Characteristics (SPC) (Medreich Plc; PL number 21880/0053).

Events <u>not</u> to be classed as SAEs on this BETTER-B Feasibility trial

The following events will **<u>not</u>** be classed as SAEs within this trial and will therefore not be subject to **<u>expedited</u>** reporting (they will still need to be reported to CTRU along with other AEs):

Hospitalisation or admission into a hospice, nursing home or palliative care unit due to:

- Care-giver burden;
- Expected deterioration related to underlying cancer diagnosis;
- Expected deterioration related to underlying lung disease diagnosis (COPD / ILD);
- Expected deterioration related to underlying chronic heart failure diagnosis (e.g. acute decompensation of heart failure, angina with or without raised troponins, cardiac arrhythmia Routine treatment or monitoring of the studied indication not associated with any deterioration in condition;

Higginson IJ, *et al. Thorax* 2020; 75:176–179. doi: 10.1136/thoraxjnl-2019-213879

- Treatment which was elective or pre-planned, for a pre-existing condition not associated with any deterioration in condition, e.g. pre-planned hip replacement operation which does not lead to further complications;
- Any admission to hospital or other institution for general care where there was no deterioration in condition;
- Treatment on an emergency, outpatient basis for an event **not** fulfilling any of the definitions of serious as given above and not resulting in hospital admission.

Events classed as expected SAEs / SARs

Examples of events which will be classed as **expected** SAEs / SARs within this trial are given below. These will **not** be reportable as SUSARs on the trial, unless the severity of the event is considered to be unexpected.

This is not intended to be an exhaustive list, therefore when determining whether a SAE / SAR is expected or not, the pharmacovigilance reference copy of the mirtazapine SPC (Medreich Plc; PL number 21880/0053) must always be referred to.

Examples of expected SARs (related to mirtazapine):

- Increase in appetite
- Weight gain
- Somnolence
- Sedation
- Headache
- Dry mouth

All events should be reviewed and classified by the site PI, or another clinically qualified member of the medical team authorised in the BETTER-B Delegation Log.

Higginson IJ, *et al. Thorax* 2020; 75:176–179. doi: 10.1136/thoraxjnl-2019-213879

1.17.2 Suspected Unexpected Serious Adverse Reactions (SUSARs)

For general a definition of SUSARs, please see section 1.16 above.

SUSARs and will be subject to expedited reporting to the Medicines and Healthcare products Regulatory Authority (MHRA) and Research Ethics Committee (REC).

Events associated with placebo will usually not satisfy the criteria for a SUSAR and therefore expedited reporting. However, where SARs are thought to be associated with placebo (e.g. reaction due to excipient or impurity) the CTRU will report such cases to the CI for assessment of expectedness and, if appropriate, to the Sponsor for onward reporting to the MHRA and REC.

Routinely breaking the blind could compromise the integrity of the trial. For this reason blind-breaking will only take place where information about the participant's trial treatment is clearly necessary for the appropriate medical management of the participant. In all cases the Investigator would be expected to evaluate the causality and expectedness of SAEs/SARs as though the participant was receiving the active medication.

1.18 BETTER-B Reporting Requirements

Information about all events (AEs, ARs, SAEs, SARs and SUSARs), whether volunteered by the participant, discovered by investigator questioning or detected through physical examination, laboratory test or other investigation, must be collected and reported to the CTRU.

All SAEs, SARs and SUSARs (excepting those specified in this protocol as not requiring expedited reporting) must be reported immediately (and certainly **no later than 24 hours**) by the trial site team to the CTRU.

For each SAE/SAR or SUSAR the following information will be collected:

- event duration (start and end dates, if applicable)
- action taken
- outcome
- "key information":
    - full details in medical terms and case description (or signs / symptoms / diagnosis – i.e. adequate information describing the event)
    - seriousness criteria

Higginson IJ, *et al. Thorax* 2020; 75:176–179. doi: 10.1136/thoraxjnl-2019-213879

- causality (i.e. relatedness to mirtazapine / investigation), in the opinion of the investigator
- whether the event would be considered expected or unexpected
- PI signature (or another clinically qualified member of the medical team authorised in the BETTER-B Authorised Personnel Log)

All events must be reviewed and assessed (for seriousness, causality and expectedness) by the PI, or another clinically qualified member of the medical team authorised in the BETTER-B Delegation Log. If an authorised medic is not available on the day the site team become aware of the event, initial reports without causality and expectedness must still be sent to the CTRU **within 24 hours** of the site becoming aware, and must be followed-up by medical assessment as soon as possible thereafter. Any outstanding “key information” (see above) must be reported within a further 24 hours. Subsequently, follow-up reports (detailing changes in condition) must be reported to the CTRU within 24 hours of the site becoming aware of a change relating to “key information”, or at the time of the event resolving or, for all other data, when requested by the CTRU.

1.18.1 Reporting of Adverse Events (AEs) and Adverse Reactions (ARs)

All AEs occurring **from randomisation up to 7 days after the last dose of trial treatment** and all ARs occurring **from the first trial treatment dose up to 7 days after the last dose of trial treatment** must be recorded on the appropriate Trial Treatment Assessment CRF or Follow-up Assessment CRF, which will be posted to CTRU within 2 weeks of the assessment. These are not subject to expedited reporting to CTRU.

1.18.2 Expedited Reporting of Serious Adverse Events (SAEs), Serious Adverse Reactions (SARs) and Suspected Unexpected Serious Adverse Reactions (SUSARs)

All SAEs, SARs and SUSARs (see section 1.17 above for definitions) must be recorded on the appropriate CRF (SAE or SAR) and reported to the CTRU **within 24 hours** of the local research team site staff becoming aware of the event (this includes participants who have withdrawn consent for data collection, see section 1.20). Once all resulting queries have been resolved, the original wet-ink CRF will be posted to the CTRU and a copy retained at site.

Please ensure that only one event is reported on each SAE and SAR CRF (details of multiple symptoms should be listed if they relate to the same event).

SAEs, SARs and SUSARs must be reported in an expedited manner (within 24 hours of the research team becoming aware) during the active monitoring period, which is defined as

Higginson IJ, *et al. Thorax* 2020; 75:176–179. doi: 10.1136/thoraxjnl-2019-213879

occurring **from randomisation (for SAEs) or from the first trial treatment dose (for SARs and SUSARs) up to 7 days after the last dose of trial treatment**.

If sites become aware of any SARs or SUSARs occurring after this active monitoring period, these must still be reported in an expedited manner **up until 90 days after the End of Trial**.

1.19 Responsibilities

Principal Investigator:

1. Checking for AEs and ARs when participants attend for treatment / follow-up (this may be delegated to an appropriate member of the trial team) and ensuring that AEs and ARs are recorded and reported to the CTRU in line with the requirements of the protocol.
2. Checking for SAEs when participants attend for treatment / follow-up (this may be delegated to an appropriate member of the trial team).
3. Using medical judgement in assigning seriousness, causality and expectedness using the version of the pharmacovigilance reference copy of the mirtazapine SPC (Medreich Plc; PL number 21880/0053).
4. Ensuring that all SAEs (occurring up to 7 days after a participant's last trial treatment dose) and SARs, including SUSARs (occurring up to 90 days after the End of Trial) are recorded and reported to the CTRU **within 24 hours** of becoming aware of the event and provide further follow-up information as soon as available. Ensuring that SAEs and SARs (including SUSARs) are chased with CTRU if a record of receipt is not received within 2 working days of initial reporting.
5. Ensuring that SAEs are reported to local committees in line with local arrangements.

Chief Investigator (or nominated individual):

1. Clinical oversight of the safety of patients participating in the trial, including an ongoing review of the risk / benefit.
2. Using medical judgement in assigning seriousness, causality and expectedness of SAEs where it has not been possible to obtain local medical assessment.
3. Review of specific SAEs and SARs in accordance with the trial risk assessment and protocol as detailed in the Trial Monitoring Plan.
4. Review of all events assessed as SUSARs in the opinion of the local investigator. In the event of disagreement between local assessment and the

Higginson IJ, *et al. Thorax* 2020; 75:176–179. doi: 10.1136/thoraxjnl-2019-213879

Chief Investigator (CI), local assessment will not be downgraded but the CI may add comments prior to reporting to MHRA and REC.

5. Assigning Medical Dictionary for Regulatory Activities (MedDRA) or Body System Coding to all SAEs and SARs.
6. The Chief Investigator, with input from CTRU and Sponsor, will submit a Development Safety Update Report (DSUR) relating to this trial IMP, to the MHRA and REC annually.

CTRU:

1. Central data collection and verification of AEs and ARs, SAEs, SARs and SUSARs according to the trial protocol onto a MACRO database.
2. Reporting safety information to the independent oversight committee identified for the trial (Trial Steering Committee (TSC)) according to the Trial Monitoring Plan.
3. Expedited reporting of SUSARs to the MHRA, REC and Sponsor within required timelines.
4. Notifying Investigators of SUSARs that occur within the trial.
5. Checking for (annually) and notifying Principal Investigators of updates to the Reference Safety Information for the trial.
6. Preparing standard tables and other relevant information for the DSUR in collaboration with the CI.
7. Ensuring timely submission of the DSUR to Sponsor and the REC.

Sponsor:

1. Reporting safety information to the CI, delegate or independent clinical reviewer for the ongoing assessment of the risk / benefit according to the Sponsor's Risk Assessment.
2. Ensuring timely submission of the DSUR to the MHRA.

Trial Steering Committee (TSC):

1. In accordance with the Trial Terms of Reference for the TSC, periodically reviewing unblinded overall safety data to determine patterns and trends of events or identify safety issues which would not be apparent on an individual case basis.

Higginson IJ, *et al. Thorax* 2020; 75:176–179. doi: 10.1136/thoraxjnl-2019-213879

2. Unblinded safety data would only be discussed in a closed session without blinded members of the trial team present.

Participant-reported measures

The various participant-reported measures (all of which will be administered by the researcher) of symptoms, activity, Quality of Life (QoL) and outcomes used in the BETTER-B Feasibility trial have been selected based on a national consensus statement of a National Cancer Research Institute (NCRI) Group on breathlessness [2], two systematic reviews of measures of breathlessness [73, 74], and a study estimating the size of a clinically important difference [25].

Most of these measures are brief scales, with a total of 70 participant-reported questions (at baseline), which overall (time for the questions and observation) take around 30-45 minutes to complete. This has been found acceptable in other studies [75, 76]. As part of this BETTER-B Feasibility trial we will assess which scales are suitable for a future large-scale trial based on missing data, patient acceptability and time to complete, so that the questions can be kept to a minimum in the future trial.

The participant-reported measures used in the BETTER-B Feasibility trial are:

- Numerical Rating Scale (NRS) for breathlessness: this assesses the severity of breathlessness in the previous 24 hours on a 0-10 numerical rating scale, for average, and worst [76]. It will be administered to participants at baseline and at the assessment calls/visits for days 7, 14, 21 and 28.
- Modified Medical Research Council (mMRC) dyspnoea scale:[64] this assesses the overall level of breathlessness and will be administered to participants at assessment visits for days 14 and 28.
- Chronic Respiratory Disease Questionnaire (CRQ):[xxxiii] this is a 20 item widely validated health-related quality of life questionnaire. Experiences are rated on 7-point scale ranging 1 (maximum impairment) to 7 (no impairment) [77, 78] This will be administered to participants at baseline and at the assessment visits for days 14 and 28 [74].
- EQ-5D-5L: this assesses mobility, self-care, usual activities, pain/discomfort, anxiety/depression according to three levels of severity (1=no problems; 2=some or moderate problems; 3=extreme problems), plus a Visual Analogue Scale (VAS) of current health-related quality of life, scored 0-100.[76] This will be administered to participants at baseline and at the assessment visit for days 28.

[xxxiii] Copyright University Hospitals of Leicester NHS Trust.

- Integrated Palliative care Outcome Scale (IPOS):[xxxiv] this is a brief measure for advanced disease widely validated in cancer and non-cancer. Each item is rated 0 (no problem) to 4 (overwhelming problem). This will be administered to participants at baseline and at the assessment visits for days 14 and 28 [79, 80].
- Generalized Self-Efficacy Scale (GSES):[67] this assesses optimistic self-beliefs to cope with a variety of difficult demands in life. This will be administered to participants at baseline and at the assessment visit for days 28.
- Hospital Anxiety and Depression Scale (HADS):[xxxv] this is a widely used and validated scale used to assess anxiety and depression and has validity in older people to assess change, which will be administered to participants at baseline and at the assessment visits for days 14 and 28 [81].
- Client Services Receipt Inventory (CSRI):[72] is an assessment tool where patients reported the health, voluntary and social care services received over the last four weeks at baseline and at the assessment visit for day 28 .[76]

Economic Evaluation

The economic evaluation component of this trial aims to test the feasibility of collecting cost data, with modified CSRI, and quality of life data, with EQ-5D-5L. We will develop the tailored CSRI questionnaire, considering patient understanding and care settings. It will be ideal to collect cost data by formal health care, social care and informer care separately. We will identify difficulties answering CSRI questions, if any, by checking item response rate and reading free text answers to open-ended questions.

We will calculate the summary statistics of formal and informal care costs (and social care costs, if possible) for the last four weeks at baseline and at the assessment visit for day 28.

Finally we will examine the possibility of assessing the cost-effectiveness using outcome measurements (average breathlessness severity measured by NRS for breathlessness, breathlessness mastery measured by CRQ and IPOS and Quality Adjusted Life Years (QALYs) derived from using EQ-5D-5L) at 4 weeks. We will explore if it is possible to produce a cost-effectiveness plane with the results from the cost-effectiveness analysis.

[xxxiv] Permission to use obtained from the Cicely Saunders Institute.

[xxxv] HADS copyright © R.P. Snaith and A.S. Zigmond, 1983, 1992, 1994.Record form items originally published in Acta Psychiatrica Scandinavica 67, 361–70, copyright © Munksgaard International Publishers Ltd, Copenhagen, 1983.This edition first published in 1994 by nferNelson Publishing Company Ltd (now GLAssessment Ltd),389 Chiswick High Road, London W4 4AL GL Assessment Ltd is part of the Granada Learning Group.

Higginson IJ, *et al. Thorax* 2020; 75:176–179. doi: 10.1136/thoraxjnl-2019-213879

Endpoints

1.20 Primary Endpoint

The primary endpoint is the number of patients recruited across 3 hospitals over a 12-month period. This has been chosen to determine whether a larger scale trial of the same design is feasible, when expanded to additional centres [82]. The decision to proceed to a future without further amendments will be based on the ability to recruit an average of 5 patients per month within a 12-month period (e.g. approximately 60 patients).

1.21 Secondary Endpoints

1.21.1 Feasibility

Other outcome measures of feasibility will be assessed to determine whether the design of the future large-scale trial may need to be adapted to improve recruitment or reduce attrition. Physical activity and toxicity outcomes will be used to inform the design of the future trial, however they will not be used to inform the decision as to whether or not to proceed to a future large-scale trial. These are:

- Number of patients screened for eligibility and reasons for non-eligibility
- Proportion of eligible patients randomised and reasons for non-randomisation
- Proportion of participants for which blinding is maintained
- Proportion of research assessors for which blinding is maintained
- Proportion of participants remaining on study for 28 days
- Proportion of, and reasons for, participants with missing data for trial outcomes
- Proportion of participants who would be eligible for dose escalation at 28 days
- Treatment compliance over the period

Feasibility outcome measures relating to recruitment will be assessed by the use of screening logs completed at each site.

Blinding will be assessed using the Bang Blinding index [83].

Missing data and study compliance will be assessed based on completed and received CRFs, summarised for each trial outcome measure [84].

Eligibility for dose escalation will be assessed based on breathlessness intensity at day 28 and tolerability of treatment.

Higginson IJ, *et al. Thorax* 2020; 75:176–179. doi: 10.1136/thoraxjnl-2019-213879

1.21.2 Activity

- **Key Activity endpoint:** severity of breathlessness at the assessment visit for day 28 as assessed by the NRS ("at worst" severity of breathlessness over the last 24 hours).
- Severity of breathlessness at the assessment visits/calls for days 7, 14 and 21, as assessed by NRS (average and "at worst" severity of breathlessness as assessed over the last 24 hours).
- Lower extremity functioning as assessed by the Short Physical Performance Battery (SPPB) [66] at the assessment visit for day 28.
- Opioid medication: at the assessment visits/calls for days 7, 14, 21 and 28.

1.21.3 Safety and Toxicity

- Adverse events, using the Common Terminology Criteria for Adverse Events (CTCAE) categorisation (v4) [69] as reported at the assessment visits/calls for days 7, 14, 21 and 28.
- Safety will be reported based on the occurrence of SAEs, SARs and SUSARs.
- Australia-modified Karnofsky Performance Status (AKPS) and modified Medical Research Council (mMRC) dyspnoea scale at the assessment visits for days 14 and 28.

1.21.4 Symptoms and Quality of Life

- Coping self-belief assessment as assessed by the General Self-Efficacy Scale (GSES) at the assessment visit for day 28.
- Mobility, self-care, usual activities, pain/discomfort and anxiety/depression as assessed by EQ-5D-5L at the assessment visit for day 28.
- Palliative symptoms as assessed by the Integrated Palliative care Outcome Scale (IPOS) at the assessment visits for days 14 and 28.
- Anxiety and depression as assessed by the Hospital Anxiety and Depression Scale (HADS) at the assessment visits for days 14 and 28.
- QoL as assessed by Chronic Respiratory Questionnaire (CRQ) at the assessment visits for days 14 and 28.

Higginson IJ, *et al. Thorax* 2020; 75:176–179. doi: 10.1136/thoraxjnl-2019-213879

Statistical Considerations

Sample size and planned recruitment rates

As the trial is designed to assess the feasibility of conducting a future definitive large-scale trial, a formal power calculation is not considered appropriate as effectiveness is not being formally evaluated.

The future large-scale trial would be designed to detect a minimum clinically important effect size of 0.5 in NRS (or a 1 point change) [2, 25]. With 90% power testing at the 5% two-sided significance level, approximately 90 participants per arm would be required. This sample size calculation will be revisited based on the observed variability of the primary outcome in this feasibility trial. It is expected that attrition rates will be approximately 20%, however this will be assessed within this feasibility trial. Assuming a 20% attrition rate, the future trial would require approximately 230 participants in total.

Feasibility of recruitment to a future large-scale trial of the same design will be concluded if the trial is able to recruit an average of 5 patients per month over a 12-month period, equivalent to approximately 60 patients, based on 3 recruiting sites. This equates to 1-2 patients per month, per site. The sites taking part in the feasibility trial are representative of those sites which would be involved in the future larger trial. Assuming 11 sites open to recruitment in the future trial, recruiting 1-2 patients per month each, this would mean a 230-participant trial would be expected to recruit in approximately 18 months to allow for the setup and initiation of all sites.

For this feasibility trial we plan to recruit approximately 60 patients in total (i.e. 30 patients to each treatment arm) from 3 sites in the UK over a 12-month period. Guidance on pilot study design by Browne et al [85, 86] state that at least 30 patients should be included to estimate a parameter for future sample size calculation [86]. In order to estimate the expected variability of the future large-scale trial's primary outcome measure of breathlessness ("at worst") at day 28 in the mirtazapine arm, 30 participants are required. As the future trial will be randomised, this equates to a total of 60 participants required, with 1:1 randomisation.

Statistical Analysis

1.22 General Considerations

Statistical analysis of the main feasibility trial is the responsibility of the CTRU Statisticians. The analysis plan outlined in this section gives a brief description of the statistical analyses which will be carried out at the end of recruitment and trial follow-up. A final, more detailed, statistical analysis plan will be written before any analysis is undertaken. Given that this is a feasibility trial, the analysis will require descriptive statistics rather than any formal hypothesis testing.

Higginson IJ, *et al. Thorax* 2020; 75:176–179. doi: 10.1136/thoraxjnl-2019-213879

Baseline characteristics of patients will be summarised.

Qualitative and Health Economics analyses will be the responsibility of the qualitative researcher and health economist respectively.

1.23 Analysis populations

The primary endpoint analysis will be based on the population of participants randomised within the 12-month recruitment period.

Endpoints which relate to data collected prior to randomisation will be analysed using all patients approached for entry to the study.

Analyses of safety data will be carried out on the safety population, defined as those participants receiving at least one dose of trial treatment, and will summarise participants according to the treatment actually received.

The remaining analysis will be carried out on the intention-to-treat (ITT) population defined as all participants randomised to the trial, regardless of adherence to the protocol, withdrawal of consent or losses to follow-up. Participants will be included within the treatment arm to which they were randomised.

1.24 Frequency of analysis

There are no formal analyses planned until after the trial is closed to recruitment. The analysis of the primary endpoint and all secondary endpoints will take place when all participants have been followed up for safety, i.e. 7 days after last trial treatment dose.

A Trial Steering Committee (TSC) will be set up to independently review data on safety, protocol adherence and recruitment. The TSC will review safety data for all participants entered into the trial approximately 6 months into recruitment (or as deemed appropriate by the TSC). Interim reports containing safety data, protocol adherence and recruitment will be presented to the TSC in strict confidence.

1.25 Primary Endpoint Analysis

The average number of patients recruited per month across 3 trial sites over a 12-month period will be summarised, overall and by trial site. The total number of patients recruited will be summarised by month, overall and by trial site.

Summaries will be presented overall by treatment arm, and by recruitment setting and diagnosis.

Higginson IJ, *et al. Thorax* 2020; 75:176–179. doi: 10.1136/thoraxjnl-2019-213879

1.26 Secondary Endpoint Analysis

1.26.1 Feasibility

The number of approaches to patients and randomisations in total throughout the randomisation period and per month will be summarised overall and by recruitment setting and diagnosis. The proportion of screened patients who are eligible for randomisation will be presented with corresponding 95% confidence intervals. Reasons for non-eligibility will be summarised. The proportion of eligible patients who are randomised will be presented with corresponding 95% confidence intervals. Reasons for non-randomisation will be summarised.

The proportion of participants for whom blinding is maintained will be summarised overall and by treatment arm, and also by recruitment setting and diagnosis, with corresponding 95% confidence intervals. The proportion of participants who became unblinded and the reasons for unblinding will also be presented. The blinding index for each arm will be calculated using the bang blinding method along with 95% confidence interval. The blinding index calculates the difference between the proportion of correct and incorrect “guesses”. The blinding index takes values between -1 and 1.

The proportion of participants who remain on study for 28 days, will be summarised overall and by treatment arm, and also by recruitment setting and diagnosis, with corresponding 95% confidence intervals. The proportion of participants who stop treatment early and the reasons for stopping treatment will be presented.

The proportion of participants who would be eligible for dose escalation at 28 days, will be summarised overall and by treatment arm, and also by recruitment setting and diagnosis, with corresponding 95% confidence intervals. Those participants who would have not been eligible for dose escalation will be summarised along with the reason why they were not eligible for dose escalation.

The proportion of participants with missing data for each trial outcome separately will be summarised overall and by arm, at each time point of assessment. Where available, reasons for missing data will be provided.

Treatment compliance will be summarised by the proportion of participants with dose reductions or omissions and total number of missed doses, by treatment arm. Reasons for dose reductions or omissions will also be presented.

Qualitative data will be analysed by the qualitative researcher. A separate analysis plan will be written outlining the proposed analysis.

Health Economic data will be analysed by the Health Economics researcher. A separate analysis plan will be written outlining the proposed analysis.

Higginson IJ, *et al. Thorax* 2020; 75:176–179. doi: 10.1136/thoraxjnl-2019-213879

1.26.2 Activity

Descriptive summaries of average severity of breathlessness over the last 24 hours and at worst, as assessed by NRS score, will be presented overall and by arm at each time point (baseline, days 7, 14, 21 and 28). Change in average and worst 24 hour breathlessness NRS score from baseline will also be presented with corresponding 95% confidence intervals. Average and worst breathlessness/24 hours will also be presented graphically using line graphs.

Differences in average and worst breathlessness/24 hours at day 28 between arms will be estimated using multi-level repeated measures modelling adjusting for NRS score at baseline, days 7, 14 and 21, and for minimisation factors, and incorporating time, treatment, and treatment by time interaction terms. Covariate estimates will be presented with corresponding standard errors. Treatment effect size (change in average and worst breathlessness) will be presented with corresponding 95% confidence intervals.

Mean Chronic Respiratory Questionnaire (CRQ) total score will be summarised with corresponding 95% confidence intervals and presented by treatment arm for baseline, days 14 and 28 by treatment arm. Mean change from baseline will also be summarised.

Mean lower extremity functioning, measured by the Short Physical Performance Battery (SPPB) at baseline and on day 28 will be summarised with corresponding 95% confidence intervals and presented by treatment arm. Mean change from baseline will also be summarised.

Opioid medication

The proportion of participants receiving opioid medication at each visit (days 7, 14, 21 and 28) will be summarised along with the type of medication by treatment arm.

1.26.3 Safety and Toxicity

The number of SAEs, SARs and SUSARs will be summarised descriptively by arm, by causality, seriousness, and body system.

The proportion of participants experiencing each toxicity will be summarised by maximum NCI CTCAE grade experienced over 28 days, by treatment arm.

The change in AKPS and mMRC from baseline to day 14 and 28 will be summarised.

Higginson IJ, *et al. Thorax* 2020; 75:176–179. doi: 10.1136/thoraxjnl-2019-213879

1.26.4 Quality of Life and Patient-reported outcomes

The percentage of non-responders and missing data will be summarised for each measurement and time-point, overall, by treatment arm and will include the proportion of expected patient-completed questionnaire packs that are missing, the proportion with missing questionnaires from each pack, the proportion of questionnaires with missing item level data, the number of missing items on each questionnaire and the number of missing scores due to missing individual question responses (items).

Outcome measures relating to Quality of Life and patient-reported outcomes (GSES, EQ-5D-5L, IPOS, HADS) will be summarised by point estimates and 95% confidence intervals and presented by treatment arm, at each time point collected.

The mean score of the GSES will be presented by treatment arm along with standard deviations and 95% confidence intervals. The change in mean score from baseline to day 28 will also be presented.

The proprtion of particiapants reporting each level of percieved problems will be presented for the EQ-5D-5L by domain and treatment arm for day 28.

The mean IPOS score will be presented overall and for each domain by treatment arm along with 95% confidence intervals for each visit.

HADS scores will be calculated for each patient and the proportion of participants in each level of Anxiety and Depression will be presented by treatment arm for day 28.

Trial Monitoring

1.27 Trial Steering Committee

A Trial Monitoring Plan will be developed and agreed by the Trial Management Group (TMG) and Trial Steering Committee (TSC) based on the trial risk assessment; this will include on site monitoring by Sponsor.

The independent TSC will review the safety and ethics of the study. Detailed un-blinded reports will be prepared by the CTRU for the TSC approximately 6 months into recruitment, and then at the end of recruitment. The TSC will be provided with detailed unblinded reports containing the information agreed in the data monitoring analysis plan.

Any unblinded interim reports provided to the TSC will be provided by the CTRU Trial Statistician for consideration in a closed session and the reports will be securely password-protected.

Higginson IJ, *et al. Thorax* 2020; 75:176–179. doi: 10.1136/thoraxjnl-2019-213879

1.28 Data Monitoring

Due to the feasibility nature of this trial, which has no planned interim analyses or review of activity data, a separate Data Monitoring & Ethics Committee (DMEC) has not been established. Independent data and ethical monitoring activities will be conducted by the TSC as described above. For any subsequent future large-scale trial however, both a DMEC and a TSC would be established.

Data will be monitored for quality and completeness by the CTRU. Missing data will be chased until it is received, confirmed as not available, or the trial is at analysis. However, missing data items will not be chased from participants. The Investigator(s) will permit trial-related monitoring, audits, REC review, and regulatory inspections by providing Sponsor, Regulators and REC direct access to source data and other documents (e.g. patients' case sheets, blood test reports, X-ray reports, histology reports etc.).

1.29 Clinical Governance Issues

To ensure responsibility and accountability for the overall quality of care received by participants during the study period, clinical governance issues pertaining to all aspects of routine management will be brought to the attention of the TSC and, where applicable, to individual NHS Trusts.

Quality Assurance, Ethical and Regulatory Considerations

1.30 Quality Assurance

Monitoring of this trial will be to ensure compliance with GCP and scientific integrity will be managed and oversight retained, by the Sponsor Quality Team.

1.31 Serious Breaches

CTRU and Sponsor have systems in place to ensure that serious breaches of GCP or the trial protocol are picked up and reported. Investigators are required to promptly notify the CTRU of a serious breach (as defined in Regulation 29A of the Medicines for Human Use (Clinical Trials) Regulations 2004 and amendments) that they become aware of. A 'serious breach' is a breach which is likely to effect to a significant degree –

(a) the safety or physical or mental integrity of the subjects of the trial; or

(b) the scientific value of the trial.

Higginson IJ, *et al. Thorax* 2020; 75:176–179. doi: 10.1136/thoraxjnl-2019-213879

In the event of doubt or for further information, the Investigator should contact the Senior Trial Co-ordinator at the CTRU.

1.32 Ethical and Regulatory Considerations

The trial will be conducted in compliance with the principles of the Declaration of Helsinki (1996), the principles of GCP and in accordance with all applicable regulatory requirements including but not limited to the NHS Research Governance Framework and the Medicines for Human Use (Clinical Trial) Regulations 2004, as amended in 2006 and any subsequent amendments. Informed written consent will be obtained from the patients prior to randomisation into the trial. The right of a patient to refuse participation without giving reasons must be respected. The participant must remain free to withdraw at any time from the trial without giving reasons and without prejudicing his/her further treatment. The study will be submitted to and approved by a REC, the MHRA for Clinical Trial Authorisation and the appropriate Site Specific Assessor for each participating trial site prior to entering patients into the trial. The CTRU will provide the REC with a copy of the final protocol, patient information sheets, consent forms and all other relevant study documentation.

The Chief Investigator will submit a final report at conclusion of the trial to the Sponsor and the REC, and the Sponsor will upload this report to the EudraCT website and notify the MHRA, within the timelines defined in the Regulations.

Confidentiality

All information collected during the course of the trial will be kept strictly confidential. Information will be held securely on paper and electronically at the CTRU and at Sponsor offices. The CTRU and Sponsor will comply with all aspects of the 1998 Data Protection Act and operationally this will include:

- consent from participants to record personal details including name, date of birth and NHS number;
- appropriate storage, restricted access and disposal arrangements for participant personal and clinical details;
- consent from participants for access to their medical records by responsible individuals from the research staff, Sponsor or from regulatory authorities, where it is relevant to trial participation;
- consent from participants for the data collected for the trial to be used to evaluate safety and develop new research;

Higginson IJ, *et al. Thorax* 2020; 75:176–179. doi: 10.1136/thoraxjnl-2019-213879

- participant name will be collected when a participant is randomised into the trial but all other data collection forms that are transferred to or from the CTRU or Sponsor will be coded with a trial number and will include two participant identifiers, usually the participant's initials and date of birth;
- where central monitoring of source documents by CTRU or Sponsor (or copies of source documents) is required (such as scans or local blood results), the participant's name must be obliterated by site before sending;
- where anonymisation of documentation is required, sites are responsible for ensuring only the instructed identifiers are present before sending to CTRU or Sponsor.

If a participant withdraws consent from further trial treatment and / or further collection of data their data collected to date will remain on file and will be included in the final study analysis.

Most CRFs will be sent to the CTRU via normal Royal Mail post, however for CRFs which need expediting to the CTRU (SAE, SAR, Death, Notification of Pregnancy, End of Trial Treatment CRFs), these must be sent either by fax or by secure encrypted electronic transfer.

For patients who take part in the Qualitative sub-study (see Appendix D), their data related to this sub-study will include audio-recordings of their interviews. This data will be collected from trial sites by the Qualitative sub-study Researcher and held at the Cicely Saunders Institute (King's College London). All data (paper and electronic) will be held securely and in accordance with the 1998 Data Protection Act.

Archiving

At the end of this trial, all trial data will be stored in line with the Medicines for Human Use (Clinical Trials) Amended Regulations 2006 and the Data Protection Act and archived in line with the Medicines for Human Use (Clinical Trials) Amended Regulations 2006 as defined in the Sponsor Archiving Standard Operating Procedure (SOP). Data held by the CTRU will be archived in the Leeds archive facility and site data and documents will be archived at the participating sites. Data held by Sponsor (on behalf of the Sponsors) on the main trial, and all Qualitative Interview data associated with the sub-study will be archived in a dedicated archive facility as designated by Sponsor. Following authorisation from Sponsor, arrangements for confidential destruction will then be made.

Higginson IJ, *et al. Thorax* 2020; 75:176–179. doi: 10.1136/thoraxjnl-2019-213879

Statement of Indemnity

The trial is sponsored by King's College London and King's College London NHS Foundation Trust. The Sponsors will at all times maintain adequate insurance in relation to the study independently. King's College London, through its own professional indemnity (Clinical Trials) and no fault compensation and the Trust having duty of care to patients via NHS indemnity cover, in respect of any claims arising as a result of clinical negligence by its employees, brought by or on behalf of a study patient.

Study Organisational Structure

1.33 Individuals and Individual Organisations

Chief Investigator (CI) – The CI is involved in the design, conduct, co-ordination and management of the trial. The CI will have overall responsibility for the design and set-up of the trial, the investigational drug supply and pharmacovigilance within the trial.

Trial Sponsor –is responsible for site monitoring, submissions to the MHRA and trial initiation management and financing of the trial as defined by Directive 2001/20/EC.

Clinical Trials Research Unit (CTRU) – The CTRU will have responsibility for conduct of the trial as delegated by Sponsor in accordance with relevant GCP standards and CTRU SOPs. The CTRU will provide set-up and monitoring of trial conduct to CTRU SOPs, and the GCP Conditions and Principles as detailed in the UK Medicines for Human Use (Clinical Trials) Regulations 2006 including, randomisation design and service, database development and provision, safety management and reporting, protocol development, CRF design, trial design and statistical analysis (excluding qualitative interview and health economic analyses) for the trial. In addition the CTRU will support REC, Site Specific Assessment and NHS Permissions submissions and clinical set-up, ongoing management including training and promotion of the trial. The CTRU will be responsible for the day-to-day running of the trial including trial administration, database administrative functions, data management and the main statistical analysis.

Central Research Nurse – The Central Research Nurse will provide support to site research nurses.

Central pharmacy – The Central pharmacy will have responsibility for trial IMP manufacture, labelling and distribution to trial sites.

Qualitative Sub-study Researcher – The Qualitative Sub-study Researcher will have responsibility for the conduct of the qualitative interview sub-study. Duties will include the training and supervision of site research teams involved in the interviews, and collection and analysis of the sub-study data.

Higginson IJ, *et al. Thorax* 2020; 75:176–179. doi: 10.1136/thoraxjnl-2019-213879

Health Economist – The Health Economist will have responsibility for the analysis of the health economy data (EQ-5D-5L and CSRI).

Higginson IJ, *et al. Thorax* 2020; 75:176–179. doi: 10.1136/thoraxjnl-2019-213879

1.34 Oversight and Trial Monitoring Groups

Trial Management Group (TMG) – The TMG, comprising the CI, Sponsor representative(s), CTRU team, other key external members of staff involved in the trial and a nursing representative will be assigned responsibility for the clinical set-up, ongoing management, promotion of the trial, and for the interpretation and publishing of the results. Specifically the TMG will be responsible for (i) protocol completion, (ii) CRF development, (iii) obtaining approval from the REC and supporting applications for Site Specific Assessments, (iv) submitting a Clinical Trial Authorisation (CTA) and obtaining approval from the MHRA, (v) completing cost estimates and project initiation, (vi) nominating members and facilitating the TSC, (vii) reporting of serious adverse events, (viii) monitoring of screening, recruitment, treatment and follow-up procedures, (ix) auditing consent procedures, data collection, trial end-point validation and database development.

Trial Steering Committee (TSC) – The TSC will provide overall supervision of the trial, in particular trial progress, adherence to protocol, participant safety and consideration of new information. It will include an Independent Chair, not less than two other independent members and a consumer representative. The CI and other members of the TMG may attend the TSC meetings and present and report progress. It is planned that this committee will meet before the trial opens to recruitment, 6 months into the recruitment period, and then again after the end of trial recruitment.

Publication Policy

The trial will be registered with an authorised registry, according to the International Committee of Medical Journal Editors (ICMJE) Guidelines, prior the start of recruitment.

The success of the trial depends upon the collaboration of all participants. For this reason, credit for the main results will be given to all those who have collaborated in the trial, through authorship and contributorship. Uniform requirements for authorship for manuscripts submitted to medical journals will guide authorship decisions. These state that authorship credit should be based only on substantial contribution to:

- conception and design, or acquisition of data, or analysis and interpretation of data,
- drafting the article or revising it critically for important intellectual content,
- and final approval of the version to be published,
- and that all these conditions must be met (www.icmje.org).

Higginson IJ, *et al. Thorax* 2020; 75:176–179. doi: 10.1136/thoraxjnl-2019-213879

In light of this, the CI, key clinical advisors and relevant senior CTRU staff will be named as authors in any publication. In addition, all collaborators will be listed as contributors for the main trial publication, giving details of roles in planning, conducting and reporting the trial.

To maintain the scientific integrity of the trial, data will not be released prior to the first publication of the analysis of the primary endpoint, either for trial publication or oral presentation purposes, without the permission of the TSC. In addition, individual collaborators must not publish data concerning their participants which is directly relevant to the questions posed in the trial until the first publication of the analysis of the primary endpoint.

Higginson IJ, *et al. Thorax* 2020; 75:176–179. doi: 10.1136/thoraxjnl-2019-213879

References

1. Parshall, M.B., et al., *An official American Thoracic Society statement: update on the mechanisms, assessments, and management of dyspnea.* Am J Respir Crit Care Med, 2012. **185**(4): p. 435-52.
2. Dorman, S., et al., *Researching breathlessness in palliative care: consensus statement of the National Cancer Research Institute Palliative Care Breathlessness Subgroup.* Palliat Med, 2009. **23**(3): p. 213-27.
3. Dudgeon, D.J., *Managing dyspnea and cough.* Hematol Oncol Clin North Am, 2002. **16**(3): p. 557-77, viii.
4. Bausewein, C., et al., *Understanding breathlessness: cross-sectional comparison of symptom burden and palliative care needs in chronic obstructive pulmonary disease and cancer.* J Palliat Med, 2010. **13**(9): p. 1109-18.
5. Gysels, M.H. and I.J. Higginson, *The lived experience of breathlessness and its implications for care: a qualitative comparison in cancer, COPD, heart failure and MND.* BMC Palliat Care, 2011. **10**(15).
6. Malik, F.A., M. Gysels, and I.J. Higginson, *Living with breathlessness: a survey of caregivers of breathless patients with lung cancer or heart failure.* Palliat Med, 2013. **27**(7): p. 647-56.
7. Kavalieratos, D., et al., *Comparing unmet needs between community-based palliative care patients with heart failure and patients with cancer.* J Palliat Med, 2014. **17**(4): p. 475-81.
8. Solano, J.P., B. Gomes, and I.J. Higginson, *A comparison of symptom prevalence in far advanced cancer, AIDS, heart disease, chronic obstructive pulmonary disease and renal disease.* J Pain Symptom Manage, 2006. **31**(1): p. 58-69.
9. Janssen, D.J., et al., *Symptoms, comorbidities, and health care in advanced chronic obstructive pulmonary disease or chronic heart failure.* J Palliat Med, 2011. **14**(6): p. 735-43.
10. Janssen, D.J., et al., *Self-perceived symptoms and care needs of patients with severe to very severe chronic obstructive pulmonary disease, congestive heart failure or chronic renal failure and its consequences for their closest relatives: the research protocol.* BMC Palliat Care, 2008. **7**(5).
11. Beynon, T., et al., *How common are palliative care needs among older people who die in the emergency department?* BMJ Support Palliat Care, 2011. **1**(2): p. 184-88.
12. Kane, P.M., K. Vinen, and F.E. Murtagh, *Palliative care for advanced renal disease: a summary of the evidence and future direction.* Palliat Med, 2013. **27**(9): p. 817-21.
13. Murtagh, F.E., J.M. Addington-Hall, and I.J. Higginson, *End-stage renal disease: a new trajectory of functional decline in the last year of life.* Journal of the American Geriatrics Society, 2011. **59**(2): p. 304-08.
14. Bausewein, C., et al., *Individual breathlessness trajectories do not match summary trajectories in advanced cancer and chronic obstructive pulmonary disease: results from a longitudinal study.* Palliat Med, 2010. **24**(8): p. 777-86.
15. Seow, H., et al., *Trajectory performance status and symptom scores for patients with cancer during the last six months of life.* J Clin Oncol, 2011. **29**(9): p. 1151-58.
16. Frostad, A., et al., *Impact of respiratory symptoms on lung cancer: 30-year follow-up of an urban population.* Lung cancer, 2008. **60**(1): p. 22-30.
17. Frostad, A., et al., *Respiratory symptoms and long-term cardiovascular mortality.* Respir Med, 2007. **101**(11): p. 2289-96.
18. Frostad, A., et al., *Respiratory symptoms as predictors of all-cause mortality in an urban community: a 30-year follow-up.* Journal of internal medicine, 2006. **259**(5): p. 520-29.

19. Janssen, D.J., et al., *A call for high-quality advance care planning in outpatients with severe COPD or chronic heart failure.* Chest, 2011. **139**(5): p. 1081-88.
20. Barbera, L., et al., *Do patient-reported symptoms predict emergency department visits in cancer patients? A population-based analysis.* Ann Emerg Med, 2013. **61**(4): p. 427-37 e5.
21. Barbera, L., C. Taylor, and D. Dudgeon, *Why do patients with cancer visit the emergency department near the end of life?* Cmaj, 2010. **182**(6): p. 563-68.
22. Currow, D.C. and A.P. Abernethy, *Breathlessness in an age of noncommunicable diseases.* Curr Opin Support Palliat Care, 2012. **6**(2): p. 127-28.
23. Yoong, J., et al., *A Pilot study to investigate adherence to long-acting opiods among patients with advanced lung cancer.* J Palliat Med, 2013. **16**(4): p. 391-96.
24. Oxberry, S.G., et al., *Repeat dose opiods may be effective for breathlessness in chronic heart failure if given for long enough.* J Palliat Med, 2013. **16**(3): p. 250-55.
25. Johnson, M.J., et al., *Opioids for chronic refractory breathlessness: patient predictors of benficial response.* Eur Respir J, 2013. **42**(3): p. 758-66.
26. Currow, D.C., M. Ekstrom, and A.P. Abernethy, *Opioids for chronic refractory breathlessness: right patient, right route?* Drugs, 2014. **74**(1): p. 1-6.
27. Currow, D.C., et al., *Once-daily opiods for chronic dyspnea: a dose increment and pharmacovigilance study.* J Pain Symptom Manage, 2011. **42**(3): p. 388-99.
28. Currow, D.C., et al., *Can we predict which patients with refractory dyspnea will respond to opioids?* J Palliat Med, 2007. **10**(5): p. 1031-36.
29. Jennings, A.L., et al., *A systematic review of the use of opioids in the management of dyspnoea.* Thorax, 2002. **57**(11): p. 939-44.
30. Smoller, J.W., et al., *Panic anxiety, dyspnea, and respiratory disease. Theoretical and clinical considerations.* American Journal of Respiratory and Critical Care Medicine, 1996. **154**(1): p. 6-17.
31. Smoller, J.W., et al., *Sertraline effects on dyspnea in patients with obstructive airways disease.* Psychosomatics, 1998. **39**(1): p. 24-29.
32. Rampin, O., O. Pierrefiche, and M. Denavit-Saubie, *Effects of serotonin and substance P on bulbar respiratory neurons in vivo.* Brain Research, 1993. **622**(1-2): p. 185-93.
33. Papp, L.A., et al., *Sertraline for chronic obstructive pulmonary disease and comorbid anxiety and mood disorders.* The American Journal of Psychiatry, 1995. **152**(10): p. 1531.
34. Currow, D., *A phase II, double-blind, multi-site randomised controlled pilot study of sertraline compared with placebo in relieving refractory breathlessness*. 2008, Australian New Zealand Clinical Trials Registry.
35. Ben-Aharon, I., et al., *Interventions for alleviating cancer-related dyspnea: a systematic review and meta-analysis.* Acta Oncologica (Stockholm, Sweden), 2012. **51**(8): p. 996-1008.
36. Booth, S., S.H. Moosavi, and I.J. Higginson, *The etiology and management of intractable breathlessness in patients with advanced cancer: a systematic review of pharmacological therapy.* Nature Clinical Practice Oncology, 2008. **5**(2): p. 90-100.
37. Simon, S.T., et al., *Benzodiazepines for the relief of breathlessness in advanced malignant and non-malignant diseases in adults.* Cochrane database systematic reviews (Online), 2010. **1**: p. CD007354.
38. Simon, S.T., et al., *Fentanyl for the relief of refractory breathlessness: a systematic review.* J Pain Symptom Manage, 2013. **46**(6): p. 874-86.
39. Jennings, A.L., et al., *Opioids for the palliation of breathlessness in terminal illness.* Cochrane database systematic reviews 2001. **4**: p. CD002066.
40. Abernethy, A.P., et al., *Effect of palliative oxygen versus room air in relief of breathlessness in patients with refractory dyspnoea: a double-blind, randomised controlled trial.* Lancet, 2010. **376**(9743): p. 784-93.

Higginson IJ, *et al. Thorax* 2020; 75:176–179. doi: 10.1136/thoraxjnl-2019-213879

41. Schruers, K., et al., *Acute L-5-hydroxytryptophan administration inhibits carbon dioxide-induced panic in panic disorder patients.* Psychiatry Research, 2002. **113**(3): p. 237-43.
42. Perna, G., R. Cogo, and L. Bellodi, *Selective serotonin re-uptake inhibitors beyond psychiatry: are they useful in the treatment of severe, chronic, obstructive pulmonary disease?* Depression and Anxiety, 2004. **20**(4): p. 203-04.
43. Devoto, P., et al., *Mirtazapine-induced corelease of dopamine and noradrenaline from noradrenergic neurons in the medal prefrontal and occipital cortex.* Eur J Pharmacol, 2004. **487**(1-3): p. 105-11.
44. Stahl, S.M., *Psychopharmacology: neuroscientific basis and practical applications*. 3rd ed. ed. 2008, Cambridge: Cambridge University Press.
45. Watanabe, N., et al., *MIrtazapine versus other antidepressive agents for depression.* Cochrane database systematic reviews, 2011. **12**: p. CD006528.
46. Kim, J.E., et al., *Efficacy and tolerability of mirtazapine in treating major depressive disorder with anxiety symptoms: an 8-week open-lable randomised paroxetine-controlled trial.* International Journal of Clinical Practice, 2011. **65**(3): p. 323-29.
47. Rayner, L., et al., *Antidepressants for the treatment of depression in palliative care: systematic review and meta-analysis.* Palliat Med, 2011. **25**(1): p. 36-51.
48. Rayner, L., et al., *Expert opinion on detecting and treating depression in palliative care: a Delphi study.* BMC Palliat Care, 2011. **10**(10).
49. Rayner, L., et al., *The development of evidence-based European guidelines on the management of depression in palliative cancer care.* Eur J Cancer, 2011. **47**(5): p. 702-12.
50. Kim, S.W., et al., *Effectiveness of mirtazapine for nausea and insomnia in cancer patients with depression.* Psychiatry and Clinical Neurosciences, 2008. **62**(1): p. 75-83.
51. Bet, P.M., et al., *Side effects of antidepressants during long-term use in a naturalistic setting.* European Neuropsychopharmacology: The Journal of the European College of Neuropsychopharmacology, 2013. **23**(11): p. 1443-51.
52. Disler, R.T., et al., *Experience of advanced chronic obstructive pulmonary disease: metasynthesis of qualitative research.* J Pain Symptom Manage, 2014. **48**(6): p. 1182-99.
53. Ramsenthaler, C., et al., *Cognitive behavioural therapy in COPD: a systematic review and meta-analysis of its effect on anxiety, panic, breathlessness and quality of life*.
54. Dudgeon, D.J., M. Lertzman, and G.R. Askew, *Physiological changes and clinical correlations of dyspnea in cancer outpatients.* J Pain Symptom Manage, 2001. **21**(5): p. 373-39.
55. Croom, K.F., C.M. Perry, and G.L. Plosker, *Mirtazapine: a review of its use in major depression and other pyschiatric disorders.* CNS Drugs, 2009. **23**(5): p. 427-52.
56. Chen, C.C., et al., *Premedication wtih mirtazapine reduces preoperative anxiety and postoperative nausea and vomiting.* Anesthesia and analgesia, 2008. **106**(1): p. 109-13.
57. Brannon, G.E. and K.D. Stone, *The use of mirtazapine in a patient with chronic pain.* J Pain Symptom Manage, 1998. **18**(5): p. 382-85.
58. Ritzenthaler, B.M.E. and D.J. Pearson, *Efficacy and tolerability of mirtazapine in neuropathic pain.* Palliat Med, 2000. **14**(4): p. 325-51.
59. Alam, A., Z. Voronovich, and J.A. Carley, *A review of therapeutic uses of mirtazapine in psychiatric and medical conditions.* Prim Care Companion CNS Disord, 2013. **15**(5).
60. Ahmed, T., J.A. Steward, and M.S. O'Mahony, *Dyspnoea and mortality in older people in the community: a 10-year follow-up.* Age Ageing, 2012. **41**(4): p. 545-59.
61. Booth, S., *Science supporting the art of medicine: improving the management of breathlessness.* Palliat Med, 2013. **27**(6): p. 483-85.

Higginson IJ, *et al. Thorax* 2020; 75:176–179. doi: 10.1136/thoraxjnl-2019-213879

62. Clemens, K.E., M. Faust, and E. Bruera, *Update on combined modalities for the management of breathlessness.* Curr Opin Support Palliat Care, 2012. **6**(2): p. 163-67.
63. Currow, D., *A randomised, double-blind multi-site parallel arm controlled trial to assess relief of refractory breathlessness in palliative care patients taking oral sertraline versus a placebo*. 2010, Australian New Zealand Clinical Trials Registry.
64. Doherty, D.E., et al., *Chronic Obstructive Pulmonary Disease: Consensus recommendations for early diagnosis and treatment.* Journal of Family Practice, 2006. **November 2006**.
65. Guralnik, J.M., et al., *A short physical performance battery assessing lower extremity function: association with self-reported disability and prediction of mortality and nursing home admission.* J Gerontol Med Sci, 1994. **49**(2): p. M85-M94.
66. Choi, H.C., et al., *An implication of the short physical performance battery (SPPB) as a predictor of abnormal pulmonary function in aging people.* Arch Gerontol Geriatr, 2012. **54**(3): p. 448-52.
67. Schwarzer, R. and M. Jerusalem, *Generalized Self-Efficacy Scale*, in *Measures in health psychology: A user's portfolio. Causal and control beliefs*, J. Weinman, S. Wright, and M. Johnston, Editors. 1995, NFER-NELSON: Windsor, England. p. 35-37. http://userpage.fu-berlin.de/~health/selfscal.htm
68. Abernethy, A.P., et al., *The Australia-modified Karnofsky Performance Status (AKPS) scale: a revised scale for contemporary palliative care clinical practice.* BMC Palliat Care, 2005. **4**(7): p. 1-12.
69. Trotti, A., et al., *CTCAE v3.0: development of a comprehensive grading system for the adverse effects of cancer treatment.* Semin Radiat Oncol, 2003. **13**(3): p. 176-81.
70. NIfHaCE, N., *Chronic Obstructive Pulmonary Disease*. 2010, London: NICE.
71. NIfHaCE, N., *Idiopathic pulmonary fibrosis*. 2013, London: NICE.
72. Beecham, J. and M. Knapp, *Costing psychiatric interventions*, in *Measuring Mental Health Needs*, G. Thornicroft, Editor. 2001. p. 200-224.
73. Bausewein, C., et al., *Measurement of breathlessness in advanced disease: a systematic review.* Respir Med, 2007. **101**(3): p. 399-410.
74. Dorman, S., A. Byrne, and A. Edwards, *Which measurement scales should we use to measure breathlessness in palliative care? A systematic review.* Palliat Med, 2007. **21**(3): p. 177-91.
75. Booth, S., et al., *Developing a breathlessness intervention service for patients with palliative and supportive care needs, irrespective of diagnosis.* J Palliat Care, 2011. **27**(1): p. 28-36.
76. Bausewein, C., et al., *Development, effectiveness and cost-effectiveness of a new out-patient Breathlessness Support Service: study protocol of a phase III fast-track randomised controlled trial.* BMC Pulmonary Medicine, 2012. **12**(1): p. 58.
77. Puhan, M.A., et al., *Relative responsiveness of the Chronic Respiratory Questionnaire, St Georges Respiratory Questionnaire and four other health-related quality of life instruments for patients with chronic lung disease.* Respiratory Medicine, 2007. **101**(2): p. 308-16.
78. Guyatt, G.H., et al., *A measure of quality of life for clinical trials in chronic lung disease.* Thorax, 1987. **42**(10): p. 773-78.
79. Siegert, R.J., et al., *Psychological well-being and quality of care: a factor-analytic examination of the palliative care outcome scale.* J Pain Symptom Manage, 2010. **40**(1): p. 67-74.
80. Hearn, J. and I.J. Higginson, *Development and validation of a core outcome measure for palliative care: the palliative care outcome scale.* Palliat Care Core Audit Project Advisory Group; Qual Health Care, 1999. **8**(4): p. 219-27.
81. Roberts, M.H., R.B. Fletcher, and P.L. Merrick, *Tha validity and clinical utlility of the hospital anxiety and depression scale (HADS) with older New Zealanders.* Int Psychogeriatr, 2014. **26**(2): p. 325-33.

82. Lancaster, G.A., S. Dodd, and P.R. Williamson, *Design and analysis of pilot studies: recommendations for good practice.* Journal of Evaluation in Clinical Practice, 2004. **10**(2): p. 307-312.
83. Bang, H., L. Ni, and C.E. Davis, *Assessment of blinding in clinical trials.* Control Clin Trials, 2004. **25**(2): p. 143-56.
84. Kurland, B.F., L.L. Johnson, and P.H. Diehr, *Accommodation of missing data in supportive and palliative care clinical trials.* Curr Opinion Support Palliat Care, 2012. **6**(4): p. 465-70.
85. Wilson DT, Walwyn RE, Brown J, Farrin AJ, Brown SR. Statistical challenges in assessing potential efficacy of complex interventions in pilot or feasibility studies. Stat Methods Med Res. 2016;25(3):997-1009.
86. Browne, R.H., *On the Use of a Pilot Sample for Sample-Size Determination.* Statistics in Medicine, 1995. **14**(17): p. 1933-1940.
87. Sturges, J. and K. Hanrahan, *Comparing telephone and face-to-face qualitative interviewing: a research note.* Qual Res, 2004. **4**(1): p. 107-18.
88. Novick, G., *Is there a bias against telephone interviews in qualitative research?* Res Nurs Health, 2008. **31**(4): p. 391-98.
89. Hewison, J. and A. Haines *Overcoming barriers to recruitment in health research*. BMJ, 2006. **333:300**, DOI: http://dx.doi.org/10.1136/bmj.333.7562.300.
90. Gysels, M., C. Shipman, and I.J. HIgginson, *"I will do it if it will help others": motiviations among patients taking part in qualitative studies in palliative care.* J Pain Symptom Manage, 2008. **35**(4): p. 347-55.
91. Gysels, M.H., C. Evans, and I.J. Higginson, *Patient, care-giver, health professional and researcher views and experiences of participating in research at the end of life: a critical interpretive synthesis of the literature.* BMC Med Res Methodol, 2012. **12**(123).
92. Shipman, C., et al., *The views of patients with advanced cancer regarding participation in serial questionnaire studies.* Palliat Med, 2008. **22**(8): p. 913-20.
93. Newington, L. and A. Metcalfe, *Researchers' and clinicians' perceptions of recruiting participants to clinical research: a themtic meta-synthesis.* J Clin Med Res, 2014. **6**(3): p. 162-72.
94. Newington, L. and A. Metcalfe, *Factors influencing recruitment to research: qualitative study of the experiences and perceptions of research teams.* BMC Med Res Methodol, 2014. **14**(10).
95. Corbin, J. and J. Morse, *The unstructured interactive interview: issues of reciprocity and risks when dealing with sensitive topics.* Qual Inq, 2003. **9**(3): p. 335-554.

Higginson IJ, *et al. Thorax* 2020; 75:176–179. doi: 10.1136/thoraxjnl-2019-213879

Appendix A: Modified Medical Research Council (mMRC) Dyspnoea Scale

This is the modified Medical Research Council (mMRC) scale[36] that uses the same descriptors as the original MRC scale, in which the descriptors are numbered 1-5. The modified MRC scale (0-4) is used for calculation of BODE (Body mass index, airflow Obstruction, Dyspnoea and Exercise capacity) index.

Grade

0 “I only get breathless with strenuous exercise”

1 “I get short of breath when hurrying on the level or walking up a slight hill”

2 “I walk slower than people of the same age on the level because of breathlessness or have to stop for breath when walking at my own pace on the level”

3 “I stop for breath after walking about 100 yards or after a few minutes on the level”

4 “I am too breathless to leave the house” or “I am breathless when dressing”

[36] Dennis E. Doherty, MD, FCCP, Mark H. Belfer, DO, FAAFP, Stephen A. Brunton, MD Leonard Fromer, MD, Charlene M. Morris, MPAS, PA-C, Thomas C. Snader, PharmD, CGP, FASCP. Chronic Obstructive Pulmonary Disease: Consensus Recommendations for Early Diagnosis and Treatment. Journal of Family Practice, November, 2006.

Higginson IJ, *et al. Thorax* 2020; 75:176–179. doi: 10.1136/thoraxjnl-2019-213879

Appendix B: Australia-modified Karnofsky Performance Scale (AKPS)

The Australia-modified Karnofsky Performance Scale (AKPS)[37] is a measure of the patient's overall performance status or ability to perform their activities of daily living. It is a single score between 10 and 100 assigned by a clinician based on observations of a patient's ability to perform common tasks relating to activity, work and self-care. A score of 100 signifies normal physical abilities with no evidence of disease. Decreasing numbers indicate a reduced performance status. The rating should be recorded as assessed (scores in increments of 10); in between scores such as 45, 55 or scores such as 50-60 are invalid.

Here are some examples of questions you might ask the potential participant in order to assess their AKPS score:

- "Have there been any changes today with your ability to attend to activities of daily living?"
- "Are you requiring more physical care today?"
- "How much time are you actually spending in bed?"

AKPS Assessment Criteria	Score
Normal; no complaints; no evidence of disease	100
Able to carry on normal activity; minor sign of symptoms of disease	90
Normal activity with effort; some signs or symptoms of disease	80
Cares for self; unable to carry on normal activity or to do active work	70
Able to care for most needs; but requires occasional assistance	60
Considerable assistance and frequent medical care required	50
In bed more than 50% of the time	40
Almost completely bedfast	30
Totally bedfast and requiring extensive nursing care by professionals and/or family	20
Comatose or barely rousable	10
Dead	0

[37] Abernethy, A. P., Shelby-James, T., Fazekas, B. S., Woods, D. Currow, D. C. (2005). The Australia-modified Karnofsky Performance Status (AKPS) Scale: A Revised Scale for Contemporary Palliative Care Clinical Practice [Electronic Version]. BioMed Central Palliative Care, 4, 1-12

Higginson IJ, *et al. Thorax* 2020; 75:176–179. doi: 10.1136/thoraxjnl-2019-213879

Appendix C: New York Heart Association (NYHA)

Doctors usually classify patients' heart failure according to the severity of their symptoms. The table below describes the most commonly used classification system, the New York Heart Association (NYHA) Functional Classification[38]. It places patients in one of four categories based on how much they are limited during physical activity.

Class	Patient Symptoms
I	No limitation of physical activity. Ordinary physical activity does not cause undue fatigue, palpitation, dyspnea (shortness of breath).
II	Slight limitation of physical activity. Comfortable at rest. Ordinary physical activity results in fatigue, palpitation, dyspnea (shortness of breath).
III	Marked limitation of physical activity. Comfortable at rest. Less than ordinary activity causes fatigue, palpitation, or dyspnea.
IV	Unable to carry on any physical activity without discomfort. Symptoms of heart failure at rest. If any physical activity is undertaken, discomfort increases.

[38] http://www.heart.org/HEARTORG/Conditions/HeartFailure/AboutHeartFailure/Classes-of-Heart-Failure_UCM_306328_Article.jsp; accessed on 08/10/2015.

Appendix D: Qualitative Sub-study

1.35 Background

A qualitative sub-study will run alongside the BETTER-B Feasibility trial to explore participants' views of the trial, aspects that affect their willingness to participate and remain in the trial, and views of the most common effects of the treatment. This sub-study will involve qualitative interviews held with a purposively selected sample of 12-15 patients.

This sub-study will be conducted by research nurses at the participating trial sites and supported by the BETTER-B Research Associate based at Kings College London, under the supervision of the Chief Investigator Prof Higginson.

Patients may feel uncomfortable with the use of a placebo-control, or the randomisation process. Understanding why patients choose not to participate or do not take up their treatment allocation will be crucial demonstrating that recruiting to a larger scale trial is feasible. We will explore what patients understand, perceive and feel about, how the BETTER-B trial was presented to them and their expectations of trial burden. We will include those participants who have declined participation; those who agreed to participate in the trial but do not take up their treatment allocation after being randomised into a particular trial arm, and those who agree to take part. Recruitment and retention of participants is essential to demonstrate our ability to perform a definitive trial in this setting, and so this work will explore the factors influencing recruitment from the patients' perspective.

1.36 Aim

To qualitatively explore patient acceptability of the trial and recruitment processes to assist in optimisation of recruitment and follow-up strategies employed for a future large-scalerandomised controlled trial.

Objectives

- To explore patients' reasons for acceptance or refusal to participate in the BETTER-B trial
- To determine ways in which the BETTER-B trial can be improved
- To explore participants' views of the placebo-control
- To explore participants' views of the randomisation process
- To identify methods and measures to be used to help generate specific recommendations for improvement

Higginson IJ, *et al. Thorax* 2020; 75:176–179. doi: 10.1136/thoraxjnl-2019-213879

1.37 Patient Interview Eligibility Criteria

Patient Inclusion Criteria

1. Approached to consider entry into the BETTER-B Feasibility trial and **either**:
   - Agreed to participate in the trial; **or**
   - Decided against participation after randomisation; **or**
   - Decided against participation when study presented to them.
2. Willing and able to comply with requirements of this sub-study
3. Written informed consent obtained to participate in this sub-study

Patient Exclusion criteria

1. Decline participation in this sub-study
2. Unable to comply with requirements of this sub-study protocol

1.38 Sampling

We will conduct qualitative interviews with up to 15 patients (subject to data saturation). We will aim to include diverse experiences, including patients (or their families if the patients are not available) who consent and (where possible) do not consent to trial enrolment, completers and non-completers, across patients with different diseases (cancer, heart failure, COPD), different ages and ethnic groups. This sub-study will be open to patients from all BETTER-B trial sites.

Interviews will be collected after the end of the participation for patients who complete, or after non-consent or withdrawal (where ethically feasible).

1.39 Consent Process

Approaching patients who have consented to the main BETTER-B Feasibility trial

Patients who consent to the main BETTER-B Feasibility trial will be asked if they would be happy to be approached about this sub-study at the time of consent into the main trial – this is an optional consent item on the main trial's Informed Consent Form (ICF).

Approaching patients who have declined to the main BETTER-B Feasibility trial

Patients who decline to participate in the main BETTER-B Feasibility trial will be provided with a BETTER-B Feedback Questionnaire at the time of refusal. The last question on this questionnaire is about the qualitative sub-study and patients can indicate whether or not they would like to be approached about the sub-study. This questionnaire is entirely optional however, so we anticipate that some patients will complete this and therefore be able to indicate their willingness to know more about the sub-study, whereas other patients will not

Higginson IJ, *et al. Thorax* 2020; 75:176–179. doi: 10.1136/thoraxjnl-2019-213879

wish to complete the questionnaire, but may still be willing to speak to a research nurse as part of this sub-study.

For those patients who decline to participate in the main trial and agree to complete the BETTER-B Feedback Questionnaire, they can indicate that they would be happy to be approached about this qualitative sub-study by answering the last question on the Feedback Questionnaire.

For those patients who decline to participate in the main trial and also decline to complete the BETTER-B Feedback Questionnaire, they will be asked at the time of refusal if they would be happy to be approached about this qualitative sub-study.

Consent to the qualitative sub-study

Those who do consent to be approached regarding the qualitative sub-study will be contacted by a member of their trial site's BETTER-B research team who will briefly describe this sub-study, go over its objectives, and answer any questions. The patient will also be provided with a sub-study Participant Information Sheet (PIS) and ICF. If patients give their consent to be interviewed, suitable arrangements will be made. The interview will be conducted at a time and place agreeable to the patient – this may be in the patient's home, or other location of their choice.

If patients change their mind following consent, they can withdraw from the sub-study at any time (including during the conduct of the interview). In these cases, no further contact will be made by the qualitative research team.

If the patient requires more time for consideration, they may contact their trial site's research team at a later time and arrange an interview.

1.40 Interview Procedure

Since several studies [87, 88] have pointed out that there are no major differences in the results of telephone and face-to-face interviews, the participants will be invited to be interviewed either over the phone or in person, to accommodate family and professional obligations. We selected this recruitment strategy because research shows that on one hand there is no evidence that potential participants object to such a system, while on the other hand such an approach minimises response bias and potentially increases the methodological rigour of the research [89].

Interviews will be audio-recorded and interviews are expected to last 30 to 45 minutes. Interviewers will follow a topic guide and probe specifically in areas of interest, including: why they agreed to participate or not, what might increase or reduce this, views of placebo control

Higginson IJ, *et al. Thorax* 2020; 75:176–179. doi: 10.1136/thoraxjnl-2019-213879

arm, frequency of follow up interviews, best mode of contact, views of the trial, views of method and location of interviews, view of methods of data collection. The research nurses will be trained by the Qualitative sub-study Researcher (based at King's College London) in the conduct of these qualitative interviews, and the Researcher will monitor the quality of the interviews and will conduct the analysis.

The research nurses will be supported in the conduct of the qualitative interviews by the Researcher under the supervision of Prof Higginson. Qualitative data will be transcribed as soon as it is received, and prepared for analysis. The Researcher will monitor the progress of qualitative interviews and recruitment of the sub-sample according to the matrix and identify and follow up on any aspects that need to be explored further. Qualitative interviews will be completed by the end of the BETTER-B Feasibility trial's recruitment period to allow adequate time for analysis and integration.

1.41 Data analysis

The qualitative data will be audio recorded, transcribed verbatim and analysed following the framework method established by Ritchie and Spencer to identify key themes. The framework matrix will be developed using NVivo 10 software (QSR) and incorporate the interview topic guide, ideas from the existing literature [90-94] and prominent themes identified from a preliminary review of the transcripts. The transcripts will be coded line by line and additional themes entered into the matrix where necessary. The matrix will then be populated with summarised data according to participant and theme, and used to identify common and divergent issues.

1.42 Endpoints

Issues related to trial design and conduct that may be responsible for poor recruitment will be discussed with the research team to inform recruitment for the definitive trial. This may include re-design of study information, recruitment strategy, advice about presenting the study, or discussions about equipoise.

Feedback relating to the importance and timing of candidate primary and secondary endpoints and the acceptability of and feasibility of intervention blinding will be provided to the research team to inform any subsequent large-scale trial.

1.43 Ethical Considerations

"Lone worker" policy

Interviews are being conducted on a one-to-one basis between a participant and the research nurse. As the participants can choose the time and place of the interview and can opt to be

Higginson IJ, *et al. Thorax* 2020; 75:176–179. doi: 10.1136/thoraxjnl-2019-213879

interviewed in their own homes, there is some risk to the research nurse. For this reason the research teams will follow their local “lone worker” policies.

Potential distress

Recent evidence suggests that qualitative interviewing, even when using unstructured interview guides (i.e. those which are not pre-approved by the ethics committees) does not have long-term negative effect which would require psychological treatment. In fact, the participants are far more likely to experience relief after discussing distressing experiences [95]. However, it is nevertheless possible that the participant will experience distress while remembering the nature of their illness. To address this issue we will make sure that the researcher working on the sub-study will have considerable experience in qualitative research in healthcare and working with vulnerable patient populations and (s)he will be able to handle these issues sensitively.

If the researcher is not able to address participant’s distress then they will follow their local “distress protocol” which may involve the patient being referred to the local recruiting site’s counselling service.

Distress may also be cause to the researcher themselves. Where this occurs, they should again follow the local “distress protocol”.

1.44 Confidentiality

All participants in this sub-study will sign Informed Consent Forms (ICFs) – these will be held securely at trial sites (copies will not be sent to the Qualitative sub-study Researcher at King’s College London). Sub-study participants will also have their interviews audio-recorded. This electronic data will be held securely at trial sites initially and then sent to King’s College London using encrypted electronic transfer, where the recordings will then be transcribed. All data (electronic and paper) will be held securely at King’s College London in accordance with the 1998 Data Protection Act.

Higginson IJ, *et al. Thorax* 2020; 75:176–179. doi: 10.1136/thoraxjnl-2019-213879

Online Box S1- Primary, secondary, clinical activity and other endpoints assessed in the BETTER-B trial

Primary endpoint = number of patients recruited across 3 hospitals over 12 months, target of 60 patients.

Secondary feasibility endpoints included: eligibility and recruitment rates in different settings, proportion of patients on trial at 28 days and proportion of patient with missing data, eligibility for dose escalation, and treatment compliance.

Toxicity and safety was assessed by adverse events using the National Cancer Institute (NCI) Common Terminology Criteria for Adverse Events (CTCAE) categorisation (v4), and serious adverse events. (https://evs.nci.nih.gov/ftp1/CTCAE/CTCAE_4.03/Archive/CTCAE_4.0_2009-05-29_QuickReference_8.5x11.pdf)

Blinding maintained: At day 28 participants 'guessed' their treatment arm, data used to calculate the Bang Blinding Index (BBI) (with the 95% confidence interval) to assess how successful blinding had been within the trial. [1] The BBI calculates the difference between the proportion of correct and incorrect "guesses" with values -1 to 1. A 0 as a null value indicates the most desirable situation under random blinding. A positive value may imply failure in blinding above random guessing (i.e. a majority of participants guess their treatment allocation correctly), and a negative value may suggest the success of blinding or failure of blinding in the other direction (i.e. more individuals mistakenly name the alternative treatment).

Clinical activity endpoints were:

Primary - worst breathlessness previous 24 hours: "How bad has your breathlessness felt at its worst over the past 24 hours?" Numerical Rating Scale (NRS) 0 (not breathless at all) – 10 (the worst possible breathlessness)

Patients' symptoms and quality of life (QoL**)** , average breathlessness previous 24 hours "How bad has your breathlessness felt on average over the past 24 hours?" NRS, 0 (not breathless at all) – 10 (the worst possible breathlessness), the Chronic Respiratory Questionnaire (CRQ) subscale scores, Integrated Palliative Care Outcome Scale (IPOS), Hospital Anxiety and Depression Scale, Generalised Self Efficacy Scale, EQ-5D-5L, and lower extremity functioning as assessed by the Short Physical Performance Battery (SPPB). As outlined in reference[5]

Services received, costs, other treatments: Health, social and informal care use was recorded using the Client Services Receipt Inventory (CSRI). As outlined in reference[5]

Higginson IJ, *et al. Thorax* 2020; 75:176–179. doi: 10.1136/thoraxjnl-2019-213879

Online Table S1 – Baseline demographic, clinical and minimisation characteristics

Numbers are n (%) unless indicated.

	Mirtazapine n=30	Placebo n=34
Age (years), Mean (s.d.)	72.9 (7.12)	70.6 (9.43)
**Gender**		
Men	24 (80.0%)	23 (67.6%)
Women	6 (20.0%)	11 (32.4%)
**Main Diagnosis**		
Lung Disease & Cancer	2 (6.7%)	1 (2.9%)
Lung Disease & Chronic Heart Failure	0 (0.0%)	1 (2.9%)
Cancer	0 (0.0%)	1 (2.9%)
Lung Disease	27 (90.0%)	29 (85.3%)
Chronic Heart Failure	1 (3.3%)	2 (5.9%)
Lung Disease categories *(includes lung disease + other diagnosis)*	*29 (96.7%)*	*31 (91.2%)*
COPD	*20 (69.0%)*	*20 (64.5%)*
ILD	*8 (27.6%)*	*11 (35.4%)*
COPD & ILD	*1 (3.4%)*	*0 (0.0%)*
**AKPS score, Mean (s.d.)**	62.0 (9.15)	63.8 (8.88)
**Breathlessness at worst over 24 hours NRS / 10, Mean (s.d.)**	7.6 (1.25)	8.0 (1.73)
**Breathlessness on average over 24 hours NRS / 10, Mean (s.d.)**	5.4 (1.36)	5.0 (1.76)
**mMRC grade**		
Grade 3 - breathless after walking ~90 metres/few minutes on level ground	12 (40.0%)	15 (44.1%)
Grade 4 - too breathless to leave the house or when dressing	18 (60.0%)	19 (55.9%)
**HADS score**		
0-14	19 (63.3%)	21 (61.8%)
15 or above	11 (36.7%)	13 (38.2%)
**IPOS score / 17 items, Mean (s.d.)**	21.5 (8.61)	19.7 (7.23)
**EQ-5D Index, Mean (s.d)**	0.53 (0.05)	0.60 (0.03)
**EQ-VAS, Mean (s.d)**	54.3 (17.9)	53.8 (18.0)
**Total health and social care costs in the previous 3 months (£), Mean (s.d)**	2220 (577)	2007 (727)
**Receiving opioid medication**		
Yes	11 (36.7%)	10 (29.4%)
No	19 (63.3%)	24 (70.6%)
**Participant able to complete QoL measures**	30 (100.0%)	34 (100.0%)
**Help required to complete QoL and type**		
Questions read out to participant	15 (50.0%)	16 (47.1%)
Helped to complete answers	4 (13.3%)	2 (5.9%)
Other	2 (6.7%)	1 (2.9%)
Total needing help	21/30 (70.0%)	19/34(55.9%)

Higginson IJ, *et al. Thorax* 2020; 75:176–179. doi: 10.1136/thoraxjnl-2019-213879

Online Table S2 – Screening outcomes by method and diagnosis, and screened to recruitment ratio

Setting	screened	randomised	screened to recruitment ratio	mirtazapine	placebo
outpatient	98	25	3.9	11	14
inpatient	35	4	8.8	3	1
community *	45	8	5.6	4	4
data base *	203	23	8.8	11	12
other	28	4	7.0	1	3
**total**	**409**	**64**	**6.4**	**30**	**34**
**main disease**					
cancer	19	1	19.0	0	1
lung disease	366	56	6.5	27	29
CHF	15	3	5.0	1	2
cancer + lung disease	8	3	2.7	2	1
lung disease + CHF	1	1	1.0	0	1
**total**	**409**	**64**	**6.4**	**30**	**34**

** note that some of these patients had not yet received pulmonary rehabilitation, and so might not be considered on optimal treatment. They were waiting for it to be offered to them, and it was clear that they would not commence until after the trial (~5 weeks) had completed. There was variability in the availablity of pulmonary rehabilitation across our centres.*

Higginson IJ, *et al. Thorax* 2020; 75:176–179. doi: 10.1136/thoraxjnl-2019-213879

Online Figure S1- Mean (95% confidence interval) breathlessness at average over 24 hours during the 28 days of the study, by study arm

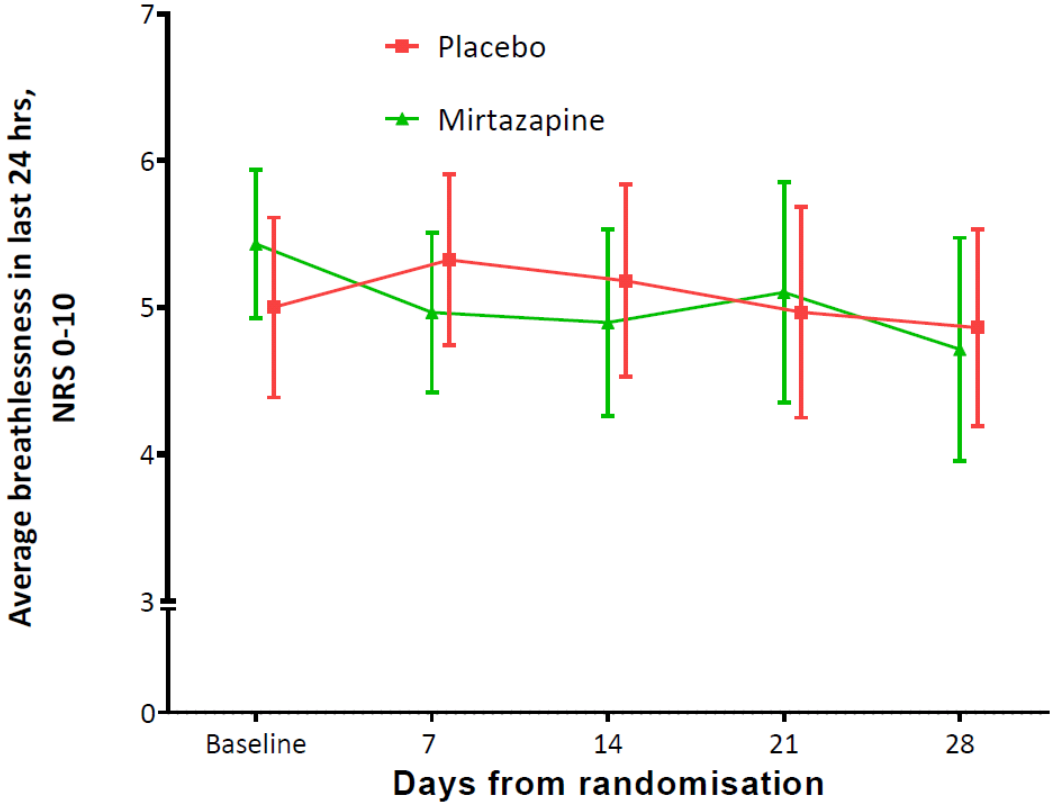

Mean (95% CI data) at each time point

Placebo	5.0 (4.4-5.6)	5.3 (4.7-5.9)	5.2 (4.5-5.8)	5.0 (4.2-5.7)	4.9 (4.2-5.5)
Mirtazapine	5.4 (4.9-5.9)	5.0 (4.4-5.5)	4.9 (4.3-5.5)	5.1 (4.4-5.9)	4.7 (4.0-5.5)

Higginson IJ, *et al. Thorax* 2020; 75:176–179. doi: 10.1136/thoraxjnl-2019-213879

Online Box S2- Qualitative data relating to analysis

Reasons for taking part

"I was approached by … during one of my regular check-ups… and I've been having problems breathing and she recommended one or two things as a result of that, and then asked if I would be interested in taking part in this study." (72 year old man, ILD)

"I took part in a previous study a few years ago, something different, and they'd asked me, cause they knew I suffer from COPD, asked me if I'd be interested." (56 year old woman, COPD).

"Because, as I say, I took that much out of the system, over- you know, and I think its time to put something back into it, you know, and if, if my condition it won't do me any good but it might help other people in the future, you know. So, my expectations are in the ways that it'll help other people in the future, you know, by me taking a part in these trials, you know, yeah. " (64 year old man, COPD)

"I was asked to take part, by the specialist… he'd been, looking after me with my problem and he suggested that I might like to take part." (84 year old man, ILD)

Home visits enabled trial participation for those who may have otherwise been unable.

"I'd have been more reluctant to take part if I had had to go to the hospital." (71 year old man, COPD)

"It could've been, shall we say, uncomfortable, getting to the hospital and getting to the clinic, if that's the word for it. Erm so it's much easier from my perspective, to be seen at home" (72 year old man, ILD)

Most participants considered the trial period of 28 days to be acceptable,

"I thought that as it was also only over a 28-day period I thought yeah, I'd, I'd be quite happy to try." (70 year old woman, ILD)

however one participant felt a slightly longer trial may be beneficial with regards to determining effect from the trial medication -

"Some days you feel healthy and some days you don't, and, the days that I feel healthy, when it's only a month's trial, it could be like, well is it the drug or isn't it the drug." (56 year old woman, COPD)

Concern re antidepressant

"That did worry me, cause I don't think I'm a depressed person…you didn't get a sheet with side effects, this is what it does, so you couldn't actually, you just, I didn't know what to expect. (72 year old man, ILD).

Higginson IJ, *et al. Thorax* 2020; 75:176–179. doi: 10.1136/thoraxjnl-2019-213879

"It wasn't a great deal of information about the actual drug, to be honest… perhaps a little more information about at the time, saying this is what the drug is and this is what, you know, its used for, it might help, I don't know" (82 year old man, Chronic Heart Failure)

Qualitative data indicated that the trial medication was straight forward to administer.

"It was tablets and I took them every day as I was asked to, um we made a note of them in a chart to make sure I had taken them, it was no problem at all." (84 year old man, ILD)

"They gave you lots of information at the beginning to say what the trial was about and, and how it would take part, length of time it will take part over, er, the number of tablets I would have to take, this was all I think about setting the base line and then I went away with the first lot of tablets" (62 year old man, COPD)

One participant did however comment on the colour and size of the capsule.

"It was bright red, which I found quite alarming- red is for danger isn't it, but I think, perhaps a gentler colour could be used… It was quite a large tablet to take along with the rest of them." (72 year old man, ILD)

Acceptability of the questionnaires, ease of completion and whether they captured aspects important to the person

"Well they were quite straightforward you know… they asked about my fitness, about my breathing, about my activities … they were straight forward." (70 year old man, COPD)

"It was based a lot on day to day things, and things that normally happened. And I think that's a really good way of people judging how much an illness is, is affecting… that's the sort of things that people like me would measure, and that's why I think the questions were realistic." (62 year old man, COPD)

The carer of one participant suggested that it could be helpful to assess impact both for the person taking the trial medication, but also for carers and those closely involved with that person. "Because what I see is not what he sees… there should be one for me, and one for him, and you should have this comparison of what the carer sees, and what the person sees" (Carer of 67 year old man, ILD)

Importance of good relationships and support for continued participation

"I saw the same nurse, yeah, she got her own case, and I saw the doctor in between, he checked you over for blood pressure and all that sort of stuff" (81 year old man, COPD)

"It was very well organised, yes, and you know, we, we had erm, information from the team and they were all great, we could always contact them if we had any problems" (82 year old man, Chronic Heart Failure)

"I felt quite safe on the trial because I was contacted every week, either with a visit, or you know, I had, 2 telephone calls I think and 2 visits" (70 year old woman, ILD)

Higginson IJ, *et al. Thorax* 2020; 75:176–179. doi: 10.1136/thoraxjnl-2019-213879

"I found that it .. was no trouble to do it at all, and that people, the people here were really good and helpful and I had a full, er, a medical before I started on the course, which was good, it eased my mind as far as that went, so, erm, yeah …. I found it was easy to do and er, just take the tablets on the night, there was no .. no inconvenience at all" (74 year old woman, COPD)<br><br>"You could talk about … your condition to them, and they didn't look down on you. They looked at you and, you know, they knew what you was talking about and … what you was going through. You know, and I could relate to what they was er, speaking about you know, and yeah I found it very helpful in one way or another'" (64 year old man, COPD)<br><br>"I felt that was good, because it was reassuring to know that there was someone who was gonna, rather than me call them, they were gonna call and say, 'are you alright' as it were. I mean, they had your telephone numbers, so I could call, but it was nice to think, well, now I've been checked on, and you're taking due care during the trial" (72 year old man ILD)

Higginson IJ, *et al. Thorax* 2020; 75:176–179. doi: 10.1136/thoraxjnl-2019-213879

Online Table S3. Missing data in the study. Categorised using the MORECare classification for missing data in palliative care studies, detailing whether item or missed questionnaire due to death or illness

Questionnaire/ outcome	Base line	MORECare Classification	Day 14	MORECare Classification	Day 28	MORECare Classification
Average NRS	0/64		1/63	2 ADI	1/58	4 ADI, 2 ADD, 1AaR
Worst NRS	0/64		1/63	2 ADI	1/58	4 ADI, 2 ADD, 1AaR
CRQ dyspnoea	0/64		2/62	3 ADI, 1 missed data item	1/58	4 ADI, 2 ADD, 1AaR
CRQ fatigue	0/64		1/62	3 ADI	1/58	4 ADI, 2 ADD, 1AaR
CRQ emotional	0/64		1/62	3 ADI	1/58	4 ADI, 2 ADD, 1AaR
CRQ mastery	0/64		1/62	3 ADI	1/58	4 ADI, 2 ADD, 1AaR
IPOS total	0/64		1/62	3 ADI	1/58	4 ADI, 2 ADD, 1AaR
IPOS pain	0/64		1/62	3 ADI	2/58	4 ADI, 2 ADD, 1AaR, 1 missed data item
IPOS shortness of breath	1/64	1 missed data item	2/62	3 ADI, 1 missed data item	1/58	4 ADI, 2 ADD, 1AaR
IPOS weakness/lack of energy	2/64	2 missed data item	2/62	3 ADI, 1 missed data item	2/58	4 ADI, 2 ADD, 1AaR, 1 missed data item
IPOS nausea	0/64		1/62	3 ADI	1/58	4 ADI, 2 ADD, 1AaR
IPOS vomiting	0/64		1/62	3 ADI	1/58	4 ADI, 2 ADD, 1AaR
IPOS poor appetite	0/64		1/62	3 ADI	1/58	4 ADI, 2 ADD, 1AaR
IPOS constipation	0/64		1/62	3 ADI	1/58	4 ADI, 2 ADD, 1AaR
IPOS sore or dry mouth	0/64		1/62	3 ADI	1/58	4 ADI, 2 ADD, 1AaR
IPOS drowsiness	0/64		1/62	3 ADI	2/58	4 ADI, 2 ADD, 1AaR, 1 missed data item
IPOS poor mobility	0/64		1/62	3 ADI	1/58	4 ADI, 2 ADD, 1AaR
IPOS anxiety	0/64		1/62	3 ADI	1/58	4 ADI, 2 ADD, 1AaR

Higginson IJ, *et al. Thorax* 2020; 75:176–179. doi: 10.1136/thoraxjnl-2019-213879

IPOS family or friend worried	0/64		1/62	3 ADI	1/58	4 ADI, 2 ADD, 1AaR
IPOS depressed	0/64		1/62	3 ADI	1/58	4 ADI, 2 ADD, 1AaR
IPOS at peace	0/64		1/62	3 ADI	1/58	4 ADI, 2 ADD, 1AaR
IPOS sharing feelings	1/64	1 missed data item	1/62	3 ADI	1/58	4 ADI, 2 ADD, 1AaR
IPOS problems addressed	0/64		1/62	3 ADI	1/58	4 ADI, 2 ADD, 1AaR
IPOS enough information	0/64		2/62	3 ADI, 1 missed data item	1/58	4 ADI, 2 ADD, 1AaR
HADS anxiety	0/64		1/62	3 ADI	1/58	4 ADI, 2 ADD, 1AaR
HADS depression	0/64		1/62	3 ADI	1/58	4 ADI, 2 ADD, 1AaR
GSES Total	0/64				2/58	4 ADI, 2 ADD, 1AaR, 1 missed data item
EQ5D mobility	0/64				1/58	4 ADI, 2 ADD, 1AaR
EQ5D self-care	0/64				1/58	4 ADI, 2 ADD, 1AaR
EQ5D usual activities	0/64				2/58	4 ADI, 2 ADD, 1AaR, 1 missed data item
EQ5D pain	0/64				1/58	4 ADI, 2 ADD, 1AaR
EQ5D anxiety and depression	0/64				1/58	4 ADI, 2 ADD, 1AaR
EQ5D health score	0/64				1/58	4 ADI, 2 ADD, 1AaR
SPPB Chair stand	11/64	11 ADI			9/58	12 ADI, 2 ADD, 1 AaR
SPPB Balance	16/64	11 ADI, 5 missing data item			15/58	12 ADI, 2 ADD, 1AaR, 6 missing data items
SPB Gait	11/64	11 ADI			9/58	12 ADI, 2 ADD, 1AaR
SPPB Summary	16/64	11 ADI, 5 missing data item			15/58	12 ADI, 2 ADD, 1AaR, 6 missing data items

ADI: attrition due to illness; ADD: attrition due to death; AaR: attrition at random

Higginson IJ, *et al. Thorax* 2020; 75:176–179. doi: 10.1136/thoraxjnl-2019-213879

Patients who missed an assessment because of being in hospital are classified as attrition due to illness

MORECare summaries account for both expected and not expected data

Higginson IJ, *et al. Thorax* 2020; 75:176–179. doi: 10.1136/thoraxjnl-2019-213879
